# Nurse-led medicines’ monitoring in care homes, implementing the Adverse Drug Reaction (ADRe) Profile improvement initiative for mental health medicines: An observational and interview study

**DOI:** 10.1371/journal.pone.0220885

**Published:** 2019-09-11

**Authors:** Sue Jordan, Timothy Banner, Marie Gabe-Walters, Jane M. Mikhail, Gerwyn Panes, Jeff Round, Sherrill Snelgrove, Mel Storey, David Hughes, on behalf of the Medicines’ Management Group, Swansea University

**Affiliations:** 1 College of Human and Health Sciences, Swansea University, Swansea, Wales, United Kingdom; 2 Cardiff and Vale University Health Board, Wales, United Kingdom; 3 Swansea Bay University Health Board, Wales, United Kingdom; 4 Institute of Health Economics, Edmonton, Alberta, Canada; 5 Hywel Dda University Health Board, Wales, United Kingdom; Nord University, NORWAY

## Abstract

**Introduction:**

Preventable adverse effects of medicines often pass unnoticed, but lead to real harm.

**Intervention:**

Nurse-led monitoring using the structured Adverse Drug Reaction (ADRe) Profile identifies and addresses adverse effects of mental health medicines.

**Objectives:**

This study investigated the implementation and clinical impact of ADRe, and barriers to and facilitators of sustained utilisation in routine practice.

**Methods:**

Administration of ADRe was observed for 30 residents prescribed mental health medicines in ten care homes. The study pharmacist reviewed completed ADRes against medication records. Policy context was explored in 30 interviews with service users, nurse managers and strategic leads in Wales.

**Results:**

Residents were aged 60–95, and prescribed 1–17 (median 9 [interquartile range (IQR) 7–13]) medicines. ADRe identified a median of 18 [IQR 11.5–23] problems per resident and nurses made 2 [1–2] changes to care per resident. For example: falls were reported for 9 residents, and care was modified for 5; pain was identified in 8 residents, and alleviated for 7; all 6 residents recognised as dyspnoeic were referred to prescribers. Nurses referred 17 of 30 residents to prescribers. Pharmacists recommended review for all 30. Doubts about administering ADRe, sometimes expressed by people who had not yet used it, diminished as it became familiar. ADRe was needed to bridge communication between resident, nurses and prescribers. When barriers of time, complacency, and doctors’ non-availability were overcome, reporting with ADRe made prescribers more likely to heed nurses’ concerns regarding residents’ welfare. Clinical gains were facilitated by one-to-one time, staff-resident relationships, and unification of documentation.

**Implications:**

To our knowledge, ADRe is the only instrument that brings a full account of patients’ problems to medication reviews. This juxtaposition of signs and symptoms against prescriptions facilitates dose adjustments and de-prescribing and leads to: reduced pain and sedation; early identification of problems linked to ADRs, such as falls; and timely medication reviews e.g. for dyspnoea.

## Introduction

Preventable adverse drug reactions and events (ADRs/ ADEs) have proved an intractable problem over the last decade, causing 5–8% of unplanned hospital admissions in the UK [1, 2] rising to ~10–15% amongst older adults [3–5], costing the UK NHS £1bn-£2.5bn each year [6]. ADRs linked to avoidable errors are responsible for 712–22,303 UK deaths each year, costing £98.5 m–£1.6bn [6]. However, higher prevalence in larger prospective studies [7], and widespread non-recognition [8] suggest that these figures may be an underestimate. Admissions related to ADRs are associated with 28–32% longer hospital stays [9] and ADRs/ADEs frequently lead to re-hospitalisation [10]. All-cause mortality is higher amongst older adults prescribed mental health medicines [11]. Demographic change and increased prescribing are likely to exacerbate ADR-related problems, and there is uncertainty over the best strategy for medicines optimisation [12].

Poor medicines’ management, including errors by patients and professionals, is largely preventable [5, 13] particularly with additional enhanced monitoring [14–19]. The Adverse Drug Reaction (ADRe) Profile [20] merged from earlier research on ADRs and nurse-led medication checking, and was designed to improve practice in this area, initially in relation to mental health medicines prescribed for care home residents [21, 22].

The ADRe Profile represents a unique approach to collating patient information and testimony to minimise ADRs, optimise prescribing, and prevent medicines-related harm and admissions. It asks nurses or carers to record the signs and symptoms of the undesirable effects of mental health medicines, as listed in manufacturers’ summaries of product characteristics (SmPCs). The supporting information suggests putative aetiologies for each sign or symptom [21]. ADRe is then shared with pharmacists and doctors reviewing medicines administration record (MAR) charts. This paper reports on implementation and operation of ADRe in a sample of Welsh care homes.

## Aims

The aims of this study were to investigate: 1) implementation of ADRe in care homes for older people, 2) the clinical impact of ADRe, including integration with pharmacists’ medication reviews, and 3) the barriers to and facilitators of sustained utilisation in routine practice.

## Methods

Ethical approval was conferred on 17th February 2017 from NHS (National Health Service) Wales Research Ethics Committee (REC) 6 (reference no. 16/WA/0358, IRAS ID 213050). The study design and methods were described in the study protocol [21] and are only summarised here, in accordance with SQUIRE 2.0 and COREQ standards (S1 File).

### Sample size

We estimated that 30 interviews and 30 observations would allow for 4–5 themes at 50% prevalence with 10 instances with 90% power, and would achieve data saturation[21].

### Inclusion criteria

We **included** residents who were: expected to remain in the home for one year; currently taking any of antipsychotics, antidepressants, anti-epileptics/ mood stabilisers, anxiolytics or hypnotics (benzodiazepines or Z drugs); willing and able to give signed informed consent themselves, or where capacity was lacking, having a consultee willing to give advice. We **excluded** those not well enough to participate, as screened by their nurses, aged <18 or receiving active palliative care [21].

**Stakeholder interviews** were conducted with study participants and key stakeholders able to give a strategic overview of the problems, including strategic leads, and the care home inspectorate [21].

### Recruitment

**Fig 1:**
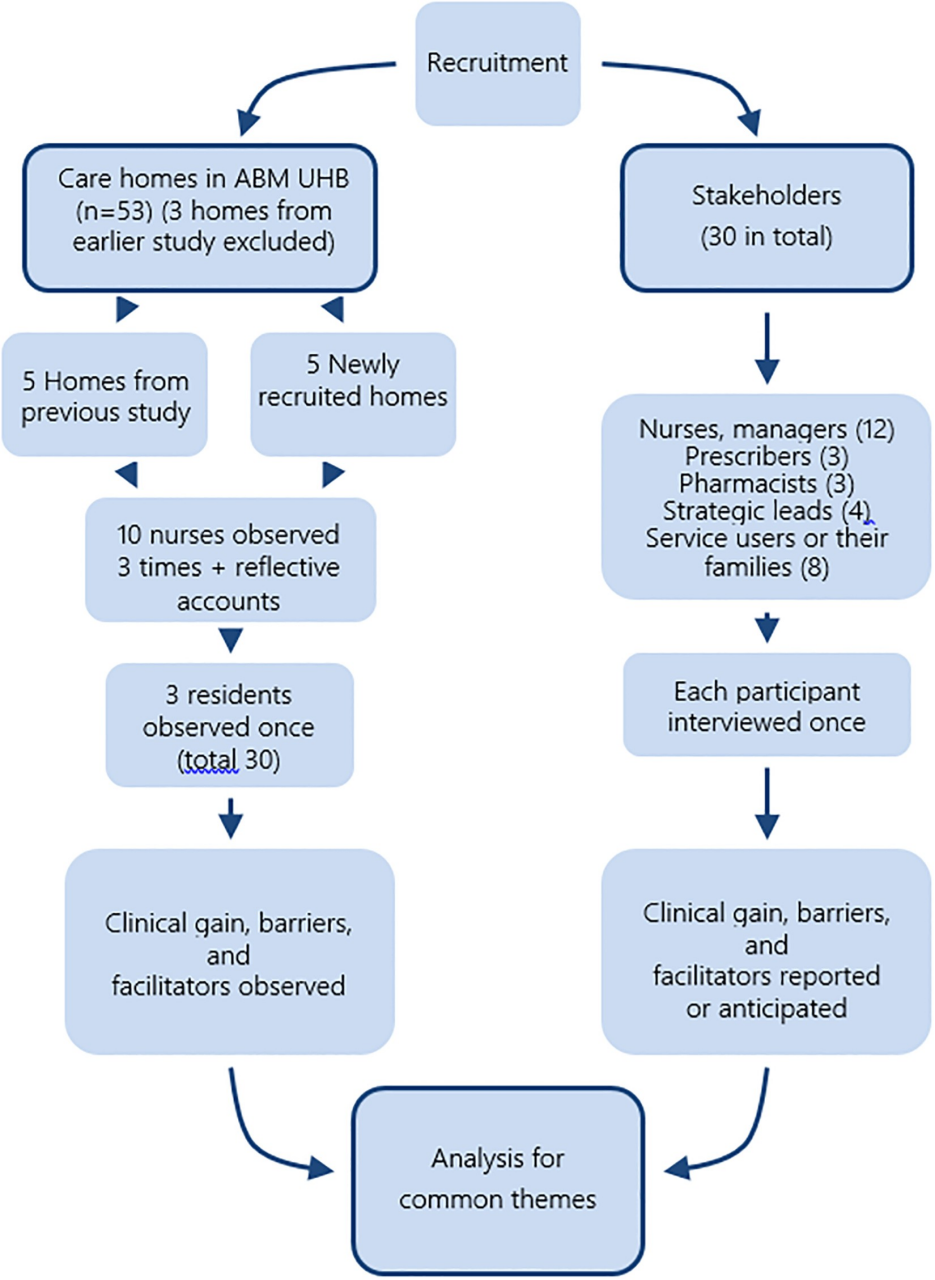
Participant flow diagram.

Volunteer care homes were sought at dissemination events and by emails sent to all 53 eligible care homes in one Welsh health board area. After initial discussion, each care home manager was sent detailed information. Residents were recruited by their nurses based on their prescriptions [21]. Three homes that had participated in the feasibility study [23], and linked with a participating home, were excluded. Professionals and service users interviewed were nurses, and where possible, residents or service users from the 10 care homes, and stakeholders identified by snowball sampling (Fig 1).

#### Setting

Administration of ADRe was observed in ten homes caring for people prescribed mental health medicines: five from our earlier randomised controlled trial [24], and five newly recruited. The wider policy context was explored in 30 interviews with service users, nurse managers and strategic leads in Wales.

### Design

This mixed-method evaluation integrated data from non-participant observation, nurses’ logs and interviews to explore how, and why ADRe works, and in what contexts [25, 26]. Debriefing and stakeholder interviews explored the problems uncovered, the role of ADRe in identifying them, changes needed to optimise clinical gain and communication between professionals.

### Outcomes

1. Implementation experiences: discrepancies between “as intended” and “as delivered”.
2. The clinical impact of ADRe was measured *via* a) problems found b) changes in processes and outcomes of care: additional care delivered, as indicated by nurses, and number and nature of clinical gains, and c) the potential for ADRe to enhance pharmacists’ medication reviews. All signs and symptoms were recorded to avoid outcome selection bias.
3. The barriers to, and facilitators of utilisation in routine practice were investigated using both observation and interview data. Contextual elements contributing to clinical gain were noted.

### Data collection

Between September 2017 and March 2018, researchers observed administration of ADRe to 30 residents in 10 care homes (SJ, MGW, SS, MS, GP) and interviewed 30 stakeholders (SJ, MGW, DH, JM, SS, MS). Data collection templates and interview questions were based on earlier work [24] and a pilot feasibility study [23] and finalised at project meetings (Tables A1 and A2 in S3 File, S2 File). Free-text fieldnotes were recorded. Interviewers were researchers with extensive experience of qualitative interviewing, with one exception, who observed earlier interviews.

#### Observations

We observed nurses’ implementation of the ADRe Profile with 30 residents, alongside standard care, within existing resources, and checked for evidence of adverse drug reactions (listed in the BNF and manufacturers’ literature). The study consultant pharmacist (TB) later reviewed the ADRe profiles alongside a copy of the corresponding MAR charts.

#### Interviews

Thirty semi-structured qualitative interviews were conducted with the main stakeholders involved in the administration of medicines in care homes: nurses and care home staff from the 10 homes (12), residents and relatives (5), prescribers (3), dispensing pharmacists (3), independent service users (3), strategic leads: Welsh policy makers (3) and a senior official from the overseeing inspectorate (1). Pharmacists and prescribers were recruited *via* PRIME centre Wales. Policy makers were recruited from the Welsh Government Health and Social Services Department. Respondents opted to be interviewed in their workplaces, homes or cafés. Interviews were audio-taped, transcribed *verbatim* and anonymised [27]. The interview schedule is appended (S2 File).

### Analysis

Demographic details, time for implementation, instances of problems identified on ADRe Profiles, and numbers and reasons for referrals were described using measures of central tendency for both normally and non-normally distributed data to facilitate consistency in reporting. Interviews and observation fieldwork were coded, categorised, analysed and closely interpreted by all authors [21]. Compliance and clinical gains were described. Interview analysis was based on the constant comparative method [28]. The two data sets were triangulated to identify common themes as described in the published protocol [21] (Fig 1). The previous themes of clinical gains, barriers, facilitators and proposals for change formed a provisional template [20–24]. Final codes and themes, as well as data saturation, reflected collective decisions.

### Ethics

The local NHS Research Ethics Committee approved the study on 17^th^ February 2017. Written and verbal information was offered and potential participants. Written informed consent was obtained for all interviews and observations. Where nurses judged that residents lacked capacity to consent, consultees signed on their behalf; consultees were relatives or professionals not involved in the study, who were in regular contact with the participants [21].

## Results

### Recruitment and retention

The research design involved data collection in ten care homes, comprising five from the previous trial [24] (100% retention) and the first five of seven ‘new’ care homes to respond positively to our invitation to 50 eligible care homes (14% response rate) (Fig 1). However, three of these latter five subsequently withdrew. These were replaced with two who had volunteered after we reached our target, plus a home approached on our behalf by a participating manager. Of the three withdrawals, one home closed, and two reported that staff found ADRe too ‘difficult’. This prompted us to interview independent service users [3].

Nurses were interviewed from all ten homes, and eight nurses returned logs of patient responses. Residents were difficult to recruit to interviews because relatively few were capable of participating, and resident or family interviews were only completed in five homes (six in all). In nine homes, all residents initially approached by their nurses consented to being observed; in the tenth, one resident declined and was replaced by an additional resident from another home. In all homes, we witnessed only care, kindness and compassion. The 30 residents observed are described in Table 1.

**Table 1:** Demographic details of the residents observed n = 30.

	Mean [SD]	Median [IQR 25^th^ to 75^th^ centile]	Range (min-max)
Age (years)	77.7 [9.9]	78 [69–85.25]	60–95
Time nurse had known resident (years)	3.8 [5.0]	2.0 [0.5–5.0]	0.25–24
Number of medicines prescribed	9.6 [4.1]	9 [7–13]	1–17
	n (%)		
Male	15 (50)		
At start of study receiving:			
• Residential care	19 (63%)		
• Nursing care	11 (37%)		

SD standard deviation, IQR interquartile range

### 1. Implementing ADRe

ADRe administration took 10–50 minutes (Table 2). Nine administrations were interrupted and distractions occurred in a further five, extending the time needed for completion. Recording vital signs took ~7 minutes. Equipment was missing for 20 observations, increasing time taken as working sphygmomanometers or thermometers were sought, sometimes in vain. Two sphygmomanometers failed to give readings from a standing position, and one nurse using previous BP recordings found only sitting BPs recorded. Given the associations between mental health medicines and hypotension, omissions of vital signs detracted from assessments of ADRs, and clinical impact. No laboratory or ECG results were available in homes (Table 3a and 3b); typically, results were logged in GPs’ surgeries. This was concerning for a man prescribed cyproterone (8.2) and more so for a woman prescribed lithium (9.1, age 93), as several problems identified might have been related to lithium (tremor, confusion, ataxia, falls and xerostomia), and perindopril, quetiapine and venlafaxine were co-prescribed.

**Table 2:** Summary of outcomes for residents (n = 30).

Observation	Mean [SD]	Median [IQR 25^th^ to 75^th^ centile]	Range (min-max)
Time for ADRe administration, including interruptions (minutes)	27.7 [12]	28.5 [18.75–40.00]	10–50
Number of problems identified / resident	17.5 [7.1]	18 [11.5–23]	6–32
Number of changes to care by nurses / resident	2.3 [1.6]	2 [1–2]	0–6
Number of pharmacist recommendations for prescription review / resident	3.8 [2.1]	3 [2–5]	1–10
Number of drug interactions* / resident	6.1 [5.7]	6 [1–9]	0–22

*using BNF drug interaction checker

**Table 3:** Potential gains identified by pharmacist review: Examples only, not a complete list.

Home	Problems on ADRe	Possible iatrogenic aetiologies	Comments, including pertinent data
1.	Dyspnoea	Asthma under-treated (beclometasone 200mcg bd, Ventolin 200mcgs inhaled qds)	Oxygen saturation was key to problem recognition.
	Postural hypotension	Quetiapine, venlafaxine, solifenacin, amlodipine,	Falls risk. ADRe informed the pharmacist that the patient had a catheter *in situ*.
	Weight gain	Mirtazapine [29]	
	Oedema	Amlodipine	
2.	Aggression	Carbamazepine, aggression exacerbated by alcohol.	
	Indigestion + constipation	Adcal D3 (contain calcium) tablets administered for indigestion.	SmPC lists these as ADRs
	Risk of falls: poor gait, confusion	Ramipril, zopiclone, carbamazepine	BP not recorded. Seizures, but no falls reported.
	Convulsions, insomnia, irritability, eczema	Levetiracetam	ADRe indicated that seizures were recurring, and only 1 AED had been prescribed.
	Feeling cold, lethargy, insomnia	Propranolol	Carers were not aware that the resident felt cold. Propranolol had been prescribed for years, but this was the first request for a review.
	Seizures	Dose of valproate low, no other anticonvulsants prescribed.	Only record of seizures was on ADRe.
	Aggression, confusion	Valproate	
	Diarrhoea, incontinence	Magnesium, valproate	The association between diarrhoea and magnesium had not been recognised.
3.	Falls	Bendroflumethiazide, ramipril, tamsulosin, zopiclone, haloperidol	Falls risk re-assessed by nurses. Postural hypotension noted for first time on ADRe.
	Resident non-ambulant	Are risendronate, calcium and folate still needed?	GP prescribing not reviewed by mental health team.
	No constipation	Senna, magnesium (2 of 3 residents)	
	Falls risk: balance/ gait / shuffling / restlessness	Ramipril, risperidone, lorazepam, diazepam, carbamazepine, furosemide	Prolonged QTc risk noted by pharmacist.
	balance/ gait / shuffling / restlessness	Risperidone	Prescriber contacted, risperidone discontinued.
4.	Shuffling, restlessness	Risperidone (2 of 3 residents)	Reviewed and discontinued.
	Postural hypotension, falls risk	Risperidone, timolol eye drops	The only documentation of standing BP was on ADRe.
	Glaucoma	Sertraline + antipsychotic	
	Weight gain	Valproate	
	Aggression	Oxazepam, valproate	Aggression was problematic for staff
	Falls	Oxazepam, valproate, zopiclone, olanzapine	
	Daytime sleeping + insomnia	Olanzapine given too early (6.00 pm.)	
	Falls (ataxia, poor gait)	Ramipril, bisoprolol, risperidone, lorazepam, carbamazepine, PRN temazepam	Sedatives reviewed and reduced.
	Insomnia	Risperidone given at night	Morning administration is advised.
	Tongue movements, shuffling, gait abnormal	Risperidone	Not previously recorded in notes.
5.	Chest pain (possibly cardiac)	Salbutamol nebules bd. + inhaler qds, chlorphenamine (above recommended dose), furosemide, prednisolone	Medicines review requested by nurse. (Digoxin co-prescribed and no potassium results located.)
	Dyspnoea	Morphine + co-codamol	Referral to respiratory nurse advised.
	Insomnia	Temazepam, Oramorph^®^, chlorphenamine, promazine given in the evening	Pharmacist advised discontinuation of all hypnotics and review of sleep hygiene.
	Pain at night	Under-treatment	Nurse to request longer-lasting analgesia e.g. transdermal preparation
	Day-time sedation	Co-codamol, chlorphenamine, morphine, temazepam PRN	Pharmacist advised discontinuation of all hypnotics and review of sleep hygiene.
	Pruritus	Emollient creams not administered, furosemide	
	Oedema	Prednisolone	
	Tremor	Promazine, salbutamol	
	Feeling cold	Bisoprolol	
	Convulsions, black outs, headaches	Citalopram antagonises phenytoin. Citalopram + paracetamol + metoclopramide risks CNS toxicity	Metoclopramide appears to be long-term (indicated for 5 days only).
	Falls, postural hypotension	Citalopram, phenytoin	ADRe offered the only documentation of standing BP.
	Mood fluctuations	Beclometasone, salbutamol	
	Hypotensive (chair bound), hypoxic	Diazepam, nitrates, sertraline, morphine	
6.	Dyspnoea / hypoxia	Possible under-treatment of COPD (ipratropium 500mcg qds, salbutamol 100mcgs qds), benzodiazepines	Prescriber contacted
	Constipation	Iron, salbutamol, ipratropium	Iron dose and formulation adjusted
	Loose teeth, dry mouth	Ipratropium	
	Bowel control	Lactulose	
	Lethargy	Atorvastatin	Statins of questionable overall value in people aged >80+ [30]
	Seizures	Carbamazepine monotherapy administered at different times each day.	Nurses previously unaware of the problems with this practice.
7.	Tremor	Valproate, levetiracetam, phenytoin	Known severe epilepsy.
	Seizures	Valproate and phenytoin	
	Hypotensive, tachycardia, hypoxic, unable to stand	Amlodipine, baclofen, oxycodone, doxazosin, furosemide, losartan, fluoxetine.	BP and oxygen saturation not routinely monitored. Pharmacist requested this. No response to intake questions.
8.	Falls / dizziness, abnormal movements / ataxia	Mirtazepine, memantine, tamsulosin (BP drop), diazepam, venlafaxine, quetiapine	Quetiapine not recommended for people with dementia.
	Mood problems	Mirtazapine, simvastatin, lansoprazole	
	Tongue movements/ abnormal movements / tremors / shuffling / ataxia	Zuclopenthixol, valproate, lorazepam	Not previously recorded in notes
	Double incontinence	Senna, magnesium, iron	
	Falls / dizziness / gait abnormal	Risperidone, atenolol, citalopram, saxagliptin, zopiclone, diazepam, memantine and atorvastatin	Recommendations to withdraw mental health medicines. Prolonged QTc risk noted by pharmacist.
	Hallucinations (aggression and violence)	Citalopram, zopiclone, memantine, possibly amlodipine	Aggression was a problem for the staff.
9.	Falls / tremor / ataxia / dizziness	Quetiapine, lithium, venlafaxine, alprazolam, perindopril, amlodipine	BP records not available. Prolonged QTc risk noted.
	Confusion / headache	Quetiapine, lithium, venlafaxine, alprazolam, perindopril, amlodipine, lansoprazole	Complex regimen needed considerable prescriber input.
	Insomnia + daytime sedation	Risperidone administered *nocte*, diazepam *mane*, zopiclone	Timing of administration to be reviewed.
10.	Ataxia / falls risk / confusion	Olanzapine, amlodipine, ramipril, co-dydramol, valproate, wine every night	
	Hypoxia (92%)	Under-treatment of COPD (no medicines listed)	Review sought.
	Ataxia, shuffling, restlessness	Risperidone	Discontinued.

Note to Table 3:

ADRe provided reassurance that some drug interactions had not adversely affected the patient:
No convulsions despite possible antagonism between up to 3 AEDs and antipsychotics (1.3, 4.2, 4.3) No signs of bleeding despite several anti-coagulant / antiplatelet agents (1.1). However, major bleeds can occur without prodromal blood loss. Not hypotensive despite several antihypertensives (8.3).

While most questions were completed in the order on ADRe, vital signs were sometimes postponed. Where these had been recorded in the resident’s notes within the last week, they were often transferred; where vital signs were stable, this was considered acceptable by care staff. Some residents had limited comprehension and speech, and entries were sometimes based on nurses’ interpretations of their health and records, for example, of bowel movements.

### 2. Clinical impact

#### a) Problems found

Care staff identified around 17 potential problems per resident when completing ADRe (Table 2), ranging from the need for oral care to extrapyramidal signs to chest pain and oxygen saturation of 89% (Tables A1 and A2 in S3 File). They made around two changes to care per resident, with changes recorded for 27 of 30 residents. In 17 cases prescribers were contacted, mainly for ‘medication review’ to address problems identified, but usually with a specific focus, such as reducing specified mental health medicines, suspected UTI or respiratory problems. Signs and symptoms possibly related to mental health medicines were almost universal. Nearly all residents had been reviewed by dentists (23 of 30) and opticians (29 of 30) in the last year. Problems found and addressed are listed in Tables A1 and A2 in S3 File.

Possible iatrogenic aetiologies of the problems recorded on ADRe were identified by the study pharmacist and checked (Tables 2 and 3). These included up to eight medicines predisposing to falls, seizures likely exacerbated by drug interactions or irregular administration of medicines, diarrhoea where laxatives had been prescribed (for earlier constipation), insomnia where olanzapine was administered too early, at 6.00pm, under-treatment of respiratory conditions, and pruritus due to suboptimal application of prescribed emollients. Continuation of some therapies was queried, for example, statins, bisphosphonates in non-ambulant residents, and medicines for prostatic hypertrophy with catheters *in situ*. Alcohol use was identified in two residents, possibly accounting for behaviour problems that might otherwise have been attributed to carbamazepine (home 2.1).

#### b) Processes and outcomes of care

Not all problems identified on ADRe were addressed (Tables A1 and A2 in S3 File), and some were not amenable to medical intervention. Debility, insomnia, cognitive decline and confusion were related to underlying disease, but were often exacerbated by prescribed medicines. Clinical gains ranged from effective analgesia to amelioration of sedation, confusion or hallucinations on discontinuation of antipsychotics (described in Table 4). In six of eight logs returned, nurses reported contacting prescribers to seek reduction in doses of antipsychotics or sedatives. Residents were reported to be ‘brighter’ where the prescriber acquiesced (five homes).

**Table 4:** Clinical gains: How ADRe helped—Facilitators and barriers (examples).

Home	Clinical gains: how problems found on ADRe were addressed	Facilitators / gains at home level	Barriers
1.	Dyspnoeic, hypoxic resident was referred to GP for management of respiratory condition Hypertension, subsequently corrected (2 residents) Diarrhoea corrected by reduction of laxatives Falls risks detected and monitored. Dizziness and falls linked with antipsychotic—review sought Hyper-salivation addressed by hyoscine patch. Follow up monitoring identified diabetes, which was treated.	Increased monitoring: postural hypotension and oxygen saturation to be monitored as on ADRe. Profiles placed in residents’ files. Profiles to be completed monthly and mental health team engaged. Increased nurses’ awareness of side effects.	Risk of duplication, as some monitoring is already done.
2.	Urination problems identified led to UTI recognition and report to GP Worsening convulsions reported to GP. Balance poor—to be observed. Resident reported feeling cold. Extra blankets made available at night.	All information in 1 place allows nurse to focus on the person. Identification of alcohol consumption helped pharmacists review drug interactions. Profile helps to “think things through”, improves knowledge and awareness. Resident positive about ADRe and reacted unusually well to researcher. Highlighted need for vital signs, not currently documented, and medicines reviews.	Nurses unsure how ADRe fits with other documentation. Care plans simply entitled “physical and mental health”. Some problems e.g. seizures, challenging behaviour, are already documented, but without indication of medication reviews. Difficult, unresponsive, hung-over and unpopular patients. Vital signs not recorded.
3.	Regular antipsychotic administration discontinued throughout the home. PRN prescriptions available. *Mood settled*, *felt safe at night*, *slept at ease when antipsychotics discontinued*. Hallucinations improved since haloperidol reduced, and then stopped. Pain: analgesia reviewed Insomnia reduced when risperidone given *mane* throughout the home. Weight loss addressed by diet diary monitoring and finger feeding. Juxtaposition of weight gain, feeling cold and hair loss prompted testing for hypothyroidism, which was then corrected.	Passing information on gait, shuffling and balance to the prescriber led to reduction and discontinuation of antipsychotics. “Using the profile has enabled us to identify and monitor problems associated with the use of antipsychotics. Staff are able to produce documented evidence to multi-disciplinary teams, to make recommendations to improve residents’ wellbeing. Staff feel valued; improvement in self-esteem has been noted. Overall benefits of using the profile has been significantly reduced, and, in some cases, discontinued, antipsychotics. Staff have become more aware of adverse drug reactions associated with these drugs, which has improved the quality of physical health for residents, preventing unnecessary admissions to hospitals.”N3 Resident said the research “is a good thing”. Mental health team engaged and reviewing profiles.	None identified.
4.	Reduction of antipsychotics throughout the home. Nurse liaised with mental health team. Tremor and hypersalivation alleviated by reduction of antipsychotics. “Much more settled” when risperidone discontinued. Sedation, benzodiazepines reduced to PRN (x2), noted as “not required”. Insomnia: zopiclone discontinued as ineffective. Urine checked, fluids encouraged throughout the home.	Mental health team responsive. Profile identified and drew attention to tremor, swelling, cognitive decline. Residents liked the attention, welcomed the checks and felt reassured. Residents and families were pleased the nurse was taking the time to check.	Profile a bit long Problems already in care plans e.g. falls, but not linked with medicines Care plans not shared with prescribers routinely.
5.	Pain: analgesia arranged (x3) Dyspnoea, GP contacted to review and ensure nebuliser available if needed. Vision poor, optician contacted. Urine tested.	Residents have the opportunity to document requests to see GP, and offer him a list of problems. Accordingly, GP contacted to review medications of all residents Nurse wishes she had been more assertive with GP. The project identified the need to check when drugs are started. Residents described ADRe as “a very good thing”.	GP not reviewing medicines when contacted. Some clients unable to understand the questions Clients’ disabilities limit their progress Clients with learning disabilities and hearing difficulties cannot discuss meds. Client repeatedly dismantles hearing aid.
6.	Falls, agitation, slurred speech triggered GP home visit, and medicines were reduced. Constipation improved when oral iron reduced, and fluid and fruit intake increased. A laxative was requested. Swallowing difficulties and ‘lacks energy’ prompted request for transfer to liquid iron preparation. Swollen, hot, dry, itching legs prompted skin care. Sleeping problems identified for a hypoxic resident using CPAP (continuous positive airways pressure)	By identifying unrecognised problems, ADRe initiated GP referral for all residents. Structured baseline information ready for GP visit. Nurses decided medicines needed changing as the best way to address problems e.g. falls, agitation, slurred speech. Residents liked the attention.	None identified.
7.	Falls—risk assessment undertaken Hypotensive and hypoxic resident administered amlodipine (presumably for hypertension), furosemide 40mg, losartan, and an alpha blocker. Pharmacist requested repeat vital signs and review of 17 medicines with 16 potential interactions. GP review sought.	None identified by nurses.	Medicines review by GP requested, but nurse fears GP only responds to changes. Medicines not routinely reviewed.
8.	Pain—analgesia administered. Over-weight (105.5 kg). Diet diaries completed and referred to dietician. (Note, hoists and other equipment sometimes have weight restrictions.) Restless, aggressive, violent, confused, hallucinating, behaviour problems: GP medicines review sought and 2 medicines discontinued.	“ADRe helps nurses’ understanding of health conditions, medicines and their changes. It addresses training issues. (…) Good opportunity to get to know and understand someone, particularly if you are new to nursing.”N8 Families would like medication reviews.	Too many problems: “Decided not to write problems in care plan, as it would take a long time.” Problems accepted as “normal for the resident”. “People don’t have time to review”. “They won’t touch complex patients” (13–15 medicines per resident in this home). Psychiatrist unavailable SU8 Breaking tablets–doses received may be unpredictable.
9.	Knee pain identified and resolved by paracetamol administration. Weight loss after hospitalisation monitored by diet diary. Weight gain, sugar and snacking reviewed with diet diary.	“Made me feel good that I helped the resident who was in pain. (…) Gives quality time with residents, confidence, and thinking.” N9	BP only taken by district nurses when they call. No vital signs recordings available.
10.	Confusion, sedation, sleep interfering with intake, cognitive decline. Nurse contacted prescribers and pharmacists. Subsequently, antipsychotics reduced to PRN for all residents, and 3 participants no longer taking any mental health medicines. Residents noted as less drowsy. Confusion and sedation—GP referral, diazepam and zopiclone discontinued and problems ameliorated. Aggression ceased when promazine stopped. Activities introduced for challenging behaviours. Incontinence ceased when promazine stopped. Intake poor (due to sedation), missing meals. Mid arm circumference and diet diary monitored to optimise intake.	“We can look immediately at mental health medications which may be causing confusion and drowsiness. (…) ADRe identified the Epilim dose was adding to Mrs. H’s confusion; she has no seizures, and is less confused. Identified that Mr.D did not require diazepam; now more alert & occasionally speaks. (…) Identified that Mr. R’s behaviour was the same without the promazine.”N10	Vital signs recorded on other documentation in the home. These need to be transcribed or ADRe needs to be integrated with other documentation.

Some homes adopted ADRe more enthusiastically than others. Implementation appeared most successful where benefits were recognised (despite initial scepticism in some instances) and prescribers responded to contacts. Certain barriers to introduction of ADRe were evident (Table 4). Triangulation of cases and nurse feedback via in-depth interviews validated these themes (Table 5; Table B in S3 File).

**Table 5:** Clinical gains, barriers and facilitators: Triangulation of observations, profiles and interviews with case examples. Full details with prescriptions in Table B in S3 File.

Theme	Subtheme	Participant	Case reports	Extracts from Interviews
**Clinical gains**	Patient more contented following reduction of antipsychotics.	3.2 Woman, 84	When ADRe was shared with the consultant, haloperidol was reduced, then stopped. PRN was retained, but rarely used. *Mood settled*, *and 3*.*2 said she felt safe at night*, *and slept at ease*. *Underactive thyroid identified and treated*.	“We look at the daily logs and the profile when we do their monthly care plans to see if we can find a reason. (…) So when the consultant team come, we can present the case. Obviously, the doctors are only coming and seeing the clients for a very short time, 1 afternoon every 10–12 weeks, so we give them as much information as we can, especially when we are saying to them, look, we don’t think this resident requires this amount of haloperidol. If we have got the evidence, they are much more inclined to take our word for it, listen to our point of view.” N3
	Aggression and incontinence disappeared when antipsychotic discontinued.	10.2 Man, 95	*Nurse completed ADRe*, *and used it as evidence to persuade GP to discontinue antipsychotic*. *Promazine was stopped*, *and within a month a*ggression and incontinence were no longer problems.	“[He was] much brighter. He was quite drowsy when he was on the medication. But once we’d convinced the doctor, that he really didn’t need it and we could manage his behaviour, he was actually all right & he settled, there was no problem, so he didn’t need the medication after all.” N10
**Barriers**	**Time as a problem for nurses and doctors**	2.2 Woman, 63	The only records of seizures were on ADRe. ADRe documented that seizures were occurring, and was used to report to prescribers. Researchers noted levetiracetam might be contributing to seizures as well as insomnia, eczema, irritability. However, no changes were made. Vital signs not recorded, and lack of time was given as the reason. Despite lack of time, convulsions were reported to prescribers	“There is no possible practical way that one is going to get to see senior medical staff and prescribers: it’s just not going to happen. The psychiatrist relies very heavily on what the care staff are saying. This might strengthen what the care staff are able to say about medicines impact, and we can only react positively.” SU2 (family) Confirmed by nurses: “You’ve got to produce the evidence to the GP and this is ideal to produce the evidence.” N2 “I don’t think GPs have got the time. They do their best while they are there, but it’s not routine to check on every patient regularly.” SU8 (family)
	**Fit with other documentation / resistance to change**	1.1 Man, 66	Vital signs results and ADRe were passed to GP with a referral for full medication review (over a year since last review) to address hypoxia, hypotension, dyspnoea, absence of constipation (laxative prescribed). The home enhanced their monitoring.	“We do monthly observations regularly, and as and when. So it’s like duplicating what we already do, but it is more in-depth. (…) it has highlighted some of the things that you wouldn’t think about asking, like dry mouth, gait, the physical side effects. (…) there are some things that I would never have thought to look for—tongue movements.” N1 “… Integrating this [ADRe] into routines, looking at whatever else they are monitoring with a view to keeping it manageable and not distracting them from interaction with residents.” Su2
	**Prescribers**	5.1 Woman, 83	ADRe identified chest pain, dyspnoea and insomnia. However, there was no response from the GP. The pharmacist indicated that sleep would be improved by moving promazine to morning administration, reducing doses of chlorphenamine to within BNF guidelines (from 16 to 12mg/ day), discontinuing ineffective hypnotics and reviewing salbutamol nebules and inhalers as indication was not clear. An optician’s appointment was made.	“What I found here is that the GPs are not very forthcoming. Um, it’s like today, the phone call that I got from the doctor with reference this lady, I had to make a week ago, so to me that’s not good enough, really. You’ve got to book a telephone consultation, if you want to discuss someone’s medications or to get advice on medication. You speak to the receptionist that you want to speak with the GP with reference bla, bla, bla, you could be waiting two—three days. Then you get frustrated yourself really. (…) ADRe picked up 2 issues. Like I say, I spoke to the GP, they weren’t forthcoming in helping, but they shut me down, really.” N5 The resident corroborated the nurse’s request for additional input: “The tablets I’m taking, I don’t know what they are for. Bed time tablets, I take them between 9 and 10, I go to bed and I’m awake between 1 & 2. I’m taking sleeping tablets & I can’t sleep & Oromorph–I’m still not sleeping.” SU5.1
	Nurse complacency / entrapment by prior expectation	7.2 Woman, 62	ADRe recorded oxygen saturation 94%, BP 109/75, HR 104, posture abnormal, dry eyes, unable to stand. 7.2 was hypotensive and taking amlodipine (presumably for hypertension), furosemide 40mg, losartan, baclofen, oxycodone, doxazosin. Tachycardia indicated baroreceptor reflex activated & hypoxia suggested reduced tissue perfusion, possibly linked with hypotension.	“Unlikely the medicines will be changed. I am sure they won’t be changed.” N7 “We get used to that person, so we might omit some signs that we don’t actually think are noticeable (…) the profile will ask you different questions that maybe you never thought of. (…) you might see but might classify that as age. You might not actually put it down to medication.” N6
	**Overwhelmed by the system** / defeatism	5.3 Woman, 81	ADRe identified pain for the first time, and the nurse reported to GP, seeking stronger analgesia and a medication review. However, there was no response.	“To me personally, it feels like they’re old, they’re stuck in a care home, what more can they do. That’s how it comes across to me. They put them in a care home & forget about them. (…) The majority of the residents have been here now a few years and it’s quite difficult to start messing around with their medication.”N5 “A lot of people are on a lot of drugs that aren’t reviewed.” I1
	**Staff turnover** / education	2.3 Woman, 69	The pharmacist recognised that beta blockers were causing the resident to feel cold. The nurse provided extra blankets, but did not raise this with prescribers. Magnesium hydroxide was prescribed to a resident with diarrhoea. These problems could be addressed by using ADRe’s supporting information to educate nurses or carers.	“If you’ve got a whole shift system of quite considerable numbers of staff dropping and changing like this, it’s about the practical dangers of not being done consistently. (…) who carries the responsibility for making sure this is happening and making observations.” SU2 offers to complete ADRe: “I wouldn’t find it burdensome.”
**Facilitators**	**1 to 1 time**	9.2 woman, 69	Knee pain identified, which responded to paracetamol administration. [9.2 was diagnosed with psychosis & recurrent depression.]	“One of the residents we spoke to, she has pain in the R knee. She never told anybody, when we do medication, she never mentioned that before. We can use our home remedy, paracetamol.” N9
	**Interpersonal relationships**	4.1 Woman, 82	4.1 was pleased that nurses were taking time to check, felt reassured, and enjoyed the attention. Medication review was arranged. Postural hypotension and falls risk were recognised, and marked for monthly review (risperidone, timolol, sertraline).	“We are told about what’s going on. We visit regularly (…) we can always talk to staff. They [care staff] check regularly, but if that can be improved then this is good.” SU4 (family)
	**All information in 1 place**	10.3 Man, 78	Nurse liaised with doctor to reduce and de-prescribe diazepam. This removed over-sedation & confusion.	“Without using the profile, we tend to find GPs would prescribe mental health medications that weren’t really appropriate. ADRe identified you didn’t really need these on a regular basis: PRN or not at all. So for all of us at H10, it did identify that we needed to be more in contact with the GPs & say, look you know, this isn’t working. This person doesn’t need to be on risperidone etc. You can distract residents: they’re much more settled without risperidone. That’s what we found.” N10
	**Identifying unrecognised problems**	8.1 Man, 89	Pain was recognised and treated with paracetamol. Diet diaries were completed to assist weight gain management. Carer felt ADRe identified too many problems to write them all into the care plan, but found ADRe a good opportunity to get to know the resident. The pharmacist identified a number of possible causes for 8.1’s tremor and falls: mirtazapine, memantine, tamsulosin, diazepam, venlafaxine, quetiapine.	“We get used to the person. We might omit some signs that actually we don’t think are noticeable… need to take notice when you look over the profile it will ask you different questions that maybe you never thought of. Someone being on a different kind of tablet might give them tremor. You might see that but might classify that as age. You might not actually put it as medication. So this is why the profile is good. And maybe sometimes we need to remember the basics of nursing. Some of us have been doing it for so long…either we forget or we make mistakes. (…) Then what’s actually what’s more obvious than what’s in our face what’s in front of us?” N8

**Vital signs**. ADRe introduced recording of oxygen saturation, standing BP, and girth. Hypoxia was recognised as a problem in three of nine residents with hypoxia (Table 4, homes 1,5); otherwise symptoms were attributed to asthma or former smoking or marked as “no problem”. Thirteen residents were assessed for possible postural hypotension: two had a SBP fall of ≥20mmHg, and two of ≥10mmHg (3 of the 4 were prescribed antipsychotics, three antidepressants and two both). Nurses noted the need to recheck, assess risk of falls or advise residents to stand slowly. ADRe identified mental health medicines, anti-hypertensives, medicines for prostatic enlargement, and drug combinations as potential aetiologies and advised on falls (Table A1 in S3 File, Tables 3 and 4, homes 1 & 7).

**Falls**. ADRe recorded falls for nine of 30 residents (6 of whom were prescribed antipsychotics), ataxia for 14 (nine prescribed antipsychotics), dizziness for seven (five prescribed antipsychotics), and postural hypotension for four (above). Nurses amended care plans for five residents in relation to dizziness, ataxia, postural hypotension or falls (Table 4, homes 1,7), without mentioning any relationship to medicines. The pharmacist recommended medication review to address risks of falling due to factors such as ataxia, dizziness or extra-pyramidal symptoms (EPS) for 14 residents, attributable to antipsychotics (8), AEDs (5), antidepressants (6) anti-hypertensives (3), benzodiazepines or zopiclone (6), and combinations of medicines. Ten of these 14 residents were referred to prescribers by their nurses (Table 4).

**Weight**. Action regarding weight change was taken for 11 residents, and for current weight for a further two, including introduction of diet diaries for nine (Table 4, homes 3,9). The pharmacist suggested that mirtazapine and valproate might be associated with weight gain, but this was not actioned.

**Pain**. ADRe identified pain for eight residents, one worsening: seven *via* the “pain question” and one when checking for non-verbal cues. Nurses acted on seven reports, by administering analgesia, monitoring pain and seeking medical advice (Table 4, homes 3,5,8,9). One resident’s message to prescriber was “pain”.

**Dyspnoea**. ADRe identified six residents as short of breath, four of whom were prescribed respiratory medicines. The pharmacist recommended review for three of four residents treated for respiratory conditions: two had oxygen saturations ≤96%. Nurses referred all six participants to prescribers after administering ADRe (Tables A1 and A2 in S3 File, homes 1,4,5,6).

**Feeling cold**. 14 residents either said they were cold or were cold to the touch (some could not speak). Extra blankets or clothes were offered. For two residents, the pharmacist recommended review of beta blockers, but nurses had not made this connection.

**Extra-pyramidal symptoms (EPS)**. ADRe identified problems associated with EPS: 15 instances of restlessness (11 prescribed antipsychotics), 12 of feet shuffling (eight prescribed antipsychotics), nine of abnormal posture (seven prescribed antipsychotics), eight of abnormal gait (six prescribed antipsychotics), five of abnormal tongue movements (all prescribed antipsychotics). Nurses rarely acted on hand tremor (one of nine was referred to the CMHT), and there were no documented responses to tongue movements, abnormal posture or gait. Pharmacist review cited EPS as a reason to review doses of antipsychotics for nine residents, AEDs for another, and drug combinations for another.

#### c) Pharmacist reviews

By juxtaposing the problems listed on ADRe with the prescriptions issued, around three items per resident were identified for prescription review or additional monitoring. Examples appear in Tables 4 and 5. Reviews were recommended for most mental health medicines (Table 6), for example antipsychotic review was recommended for 17 of 18 residents. Potential problems included safety concerns e.g. falls or risk of falls due to ataxia, abnormal gait or EPS (8), and quality of life issues e.g. insomnia when risperidone was administered *nocte* rather than *mane* (3). Nurses referred eight of the 18, and four regimens (all from 1 home) had been changed on initial use of ADRe, before its observed use. AED review was recommended for 11 of 13 residents, to prevent falls (5), or address residents’ concerns e.g. incontinence (1): nurses had referred six. ADRe identified both worsening (resident 6.3) and breakthrough seizures (resident 2.1). Most residents prescribed antidepressants were recommended for review, sometimes to mitigate ADRs from other medicines e.g. tremor, EPS, CNS depression (3) or reduce risks of bleeding and prolonged QTc intervals (2). Benzodiazepines were contributing to several problems. No harm was identified or observed as a result of administering ADRe.

**Table 6:** Pharmacist referrals.

Number of residents	Antipsychotics	AEDs	Antidepressants	Benzodiazepines or Z drugs
Prescribed	18	13	10	14
Nurses referred	8 /18	6 /13	4 /10	5 /14
Pharmacist referred	17 /18	11 /13	8 /10	11 /14
Reasons cited by pharmacist	Weight gain (1), falls/ balance/ gait/ EPS (8), anxiety/ sedation/ confusion (3), insomnia (3), outdated medication (promazine) (1)	Weight gain (1), falls/ ataxia/ dizziness (5), EPS, including tremor (4), aggression, irritability or agitation (6), sedation (1), confusion (1), incontinence (1)	Falls or dizziness (6), eyesight (1), antagonism of AEDs (2), headache and hallucinations (1), exacerbation of tremor, EPS, CNS depression (3) Antidepressants increasing risks of bleeding and prolonged QTc intervals (2)	Falls or ataxia (6), sedation or confusion (2), incontinence (1), EPS (3), aggression (3)
Also referred for antipsychotics	-	5 /13	6 /10	9 /14
Not referred for other mental health medicines	3 /18	5 /13	1 /10	2 /14

Note: some residents were referred for >1 reason.

Although several homes acknowledged delays in medication reviews, particularly for GP prescriptions, only one (N10) of the 10 care home managers consulted pharmacists. Some mentioned annual pharmacist reviews of MAR charts. Pharmacist interviewees explained that pharmacists supplying medicines to care homes do not normally conduct medication reviews unless specifically contracted to do so by the health board.

### 3. ADRe in practice

We identified several barriers to implementation and associated clinical gains, some more surmountable than others. Barriers included time, integration with existing records and information systems, complacency, prescribers’ attitudes, access to doctors, staff education and turnover. Clinical gains from ADRe were facilitated by: allocation of one-to-one time, good staff-resident relationships, unifying pertinent supporting information into a single document, and making staff feel valued.

#### a) Barriers

**Time**. Time and conflicting work demands were the most frequently cited barriers to regular use of ADRe. “The biggest barrier is time, but having said that, once you’ve used the tool and you’ve used it regularly, it becomes second nature, so it’s not that time-consuming (N10). It’s like everything else really, slow at first and use it regularly and get quicker” (N1). However, where attention to symptoms or de-prescribing made residents more comfortable, the residents were “more settled” (3.2, 4.3), less aggressive and with reduced incontinence (10.2), less confused / sedated (10.1, 10.2, 10.4), warmer (2.3), pain-free (8.1, 9.2, 5.1, 5.2, 5.3), less constipated (6.1) and accordingly easier and more pleasant to work with (Table 4). Time taken to complete ADRe was extended by (unsuccessful) attempts to locate laboratory test results and equipment. Prescribers’ time was also identified as a problem, and ADRe might lead to more efficient use of this scarce resource (SU2).

**Integration with existing records**. Each care home had its own record system. ADRe unified existing data collection. As one informant said, more work is needed to “integrate into routines, looking at whatever else they are monitoring with a view to keeping it manageable and not distracting them from interaction with residents” (Su2).

**Prescribers**. Where prescribers engaged well with care home staff and residents, important clinical gains were made (Table 4). In some instances, nurses needed to advocate for residents to effect prescription changes. One nurse who successfully advocated for a reduction in antipsychotic prescribing said: “Pharmacists are keen to come on board, the consultants too. GPs are a difficult group. (…) they always think they know best. (…) we all know that Joe Bloggs doesn’t need the risperidone, but the GP thinks he does, so it’s getting round that sort of thinking. [… There is] resistance from the GPs because they might feel threatened, they think that nurses shouldn’t be having any input–they know best” (N10). Sometimes, prescribers refused to action problems identified: “ADRe picked up issues. Like I say, I spoke to the GP, they weren’t forthcoming in helping: they shut me down” (N5). As a result, chest pain, dyspnoea and insomnia were not addressed, despite requests for contact from residents.

**Resignation**. While some nurses effectively challenged prescribers, others had become resigned to inertia: “Unlikely the medicines will be changed. I am sure they won’t be changed” (N7). Some were disillusioned after repeated attempts to get medication reviewed: “You speak to the receptionist that you want to speak with the GP with reference bla, bla, bla, you could be waiting two- three days. Then you get frustrated” (N5). Higher-level stakeholders acknowledged that “a lot of people are on a lot of drugs that aren’t reviewed”I1.

**Education and staff turnover**. Nurses and care staff who participated soon became adept in applying ADRe, and reported that ADRe was easy to use for 27 of 30 residents. Problems with staff shortages and turnover militated against education initiatives, and managers in two homes spoke of problems in ensuring that staff had sufficient information about medicines. ADRe and supporting information attempted to fill this “education gap”, and interviewees described it as an *aide memoir* that helped collect specific information in a systematic way.

#### b) Facilitators

**Interpersonal relationships**. ADRe depended on positive relationships between residents, carers, nurses and prescribers. Both carers and residents appreciated the one- to-one care, and its contribution to patient-centred care in which residents or families could participate.

**All information in one place**. Offering prescribers a single document with problems highlighted facilitated inter-professional communication, so that: “everyone is aware of what issues are coming up with the patient, and the decisions can be made between all the healthcare professionals” (P2). One nurse said: “They [doctors, pharmacists] don’t look at the assessments and care plans and things that we do because it’s just mainly for the local health board and for us to have, in order for us to handle the residents better” (N8). ADRe offered a quick and accessible alternative to up to 20 detailed care plans, promoting clinical gains. This was a view echoed by several respondents:

“All the information in one place allows you to focus on the person. (…) I did the profile myself, (…), so I did the vital signs, wrote them all down and I looked through all this to see any of the symptoms, ticked them off. (…) it was their agitation. I discussed with the GP then” (N2).

“All the information on one piece of paper, (…), rather than going through separate pieces in separate places. (…) If the doctor comes in and says “Have you done the observations?” it’s all together in one place and you can give the details to the GP” (N9).

**Recognising problems**. Some nurses acknowledged that their familiarity with residents might blind them to problems: “We get used to the person. We might omit some signs (…) when you look over the profile it will ask you different questions that maybe you never thought of: someone being on a different kind of tablet might give them tremor. You might see that but might classify that as age. You might not actually put it as medication. So this is why the profile is good” (N8). ADRe also crystallised nurses’ clinical suspicions that medicines were not optimised, despite correct doses: “If we have got the evidence, they [doctors] are much more inclined to take our word for it, listen to our point of view” (N3). However, ADRe was seen as complex, and in the view of a few, over-complex. The comprehensiveness of the instrument was both a weakness, in terms of being too difficult for some staff, and a strength, in that “[It] puts little things together that may be missed” (N3). ADRe might allow insidious, subtle ADRs to be detected and managed, and certain hard-to-diagnose conditions (hypothyroidism, pernicious anaemia) to be recognised and treated. When compared with easier tools, ADRe “covers everything, but becomes lengthier. You won’t miss anything here, but administering this would take more time and resources” (GP2).

**Valuing staff**. ADRe works by harnessing care staff’s familiarity with patients to inform doctors of ongoing undesirable effects of medicines prescribed. The supporting information provided with the ADRe Profile suggests which medicines might be responsible, and this guidance can be used to support advocacy for review and revision: “I made notes on the profile, to lead me in the right direction. Rather than just trying to put my opinion across, I had the evidence in front of me: look this is her BP, her falls have increased–four times more. Having the supporting information to say ‘it might be this medication, so can you come and review?’” (N6.2). “What it [ADRe] tends to do, is give value doesn’t it? The staff tend to feel the information that they are gathering is of value. It improves morale. The care staff–the staff feel that what they are doing is worthwhile and they are not just ticking forms, ticking boxes and just getting filed away” (N3).

### Changing priorities: Shifting the zeitgeist

The prevalence, severity and reversibility of problems identified by ADRe indicate that change is both necessary and feasible. Respondents suggested that reducing the variation between homes, services and prescribers will reduce ADRs and ADEs, and the burdens these place on service users, staff and the NHS (Table C in S3 File). The service users were the group most dissatisfied with current practice, and the most enthusiastic advocates for ADRe, based on their personal experiences and perceived need for change in mental health medication management. Their perceptions are supported by the study pharmacist’s reviews (Table 3) and the changes brought about by implementation of ADRe (Table 4).

Changes that would help protect patients from harm associated with ADRs/ADEs were identified. These included shared access to essential information, improved staff education, adequate staffing, communication with GPs, consultants and pharmacists, and prescription reviews before repeats are issued (Table C in S3 File). ADRe made a contribution in several of these areas, providing standardised patient data, informal, experiential education, a mechanism for inter-professional communication and observations that feed into prescription reviews.

All nurses and stakeholders agreed that the paper version of ADRe needs to be developed to become compatible with digital information systems: “People won’t write it twice. (…) The questions that you have here, they are good (…) the only problem is we already have a system in place.” N8 (Table C in S3 File). Integrating ADRe with electronic patient records would allow a number of tangible improvements, such as cross-population of the ADRe Profile from other records (obviating the need for any duplication of recording), selective retrieval of relevant information (e.g. positive responses only), examination of trends over time, and an online link to interaction checkers. Developing an electronic version of ADRe appears a more useful path for development than any effort to further shorten the instrument. The latter would risk detracting from “the little things” that made ADRe comprehensive: “I don’t really know if it could be condensed” (N3).

## Discussion

Patient-centred medication review is essential. ADRe’s evolving evidence base indicates it can be effective and acceptable in supporting reviews before repeat prescribing [22–24]. This study indicates that ADRe brings several benefits: it relieves residents’ ADR burden of confusion, sedation and EPS; alleviates falls, pain, and dyspnoea; optimises seizure control; and bridges communication between residents, nurses and prescribers. However, to overcome the barriers of time, education and staff turnover (only some homes), prescribers’ availability and complacency, introducing ADRe into routine practice throughout the sector will need support from regulators. Systematic, routine ADR monitoring needs to be integrated into existing care and documentation. Where prescribers visited regularly, this was relatively straightforward; however, where doctors were uncontactable, it was difficult.

### ADRs: An everyday problem in need of policy intervention

Meta-analyses identified little evidence for clinical gains from interventions to optimise prescribing [31–33] or polypharmacy [34], or physical health, (except individualised exercise interventions) [35]. While many interventions [36–40] improve medication appropriateness, links to patient outcomes are unclear [12, 31, 41, 42] *ADRe is different* [21]- it addresses the “little” things affecting patients, as a preventive strategy, for example, by addressing dizziness, balance and eyesight before falls happen (homes 1,2,6,7, Table 4) [43, 44].

ADRs and ADEs are common, though often unrecognised [8], and systematic checking with ADRe increased recognition, reporting and actions [20, 24] Preventable problems were almost universal, but often mundane, commonplace, un-recorded, and, consequently, overlooked. ADRe helped to break the Inverse Interest Law: the commoner the problem, the less the professional interest [45]. Without ADRe, clinically uninteresting, yet debilitating, conditions, such as incontinence, confusion, xerostomia, hyper-salivation, and insomnia were often under-investigated (all homes Table 4).

The differences in medical care available to residents in different homes, particularly access to consultants [46], reflected the association between socio-economic deprivation and GP scarcity [47], and the Inverse Care Law [48]. Without change in policy, homes in the poorest areas will be the least able to adopt ADRe, because they cannot contact prescribers to address problems identified (homes 5 & 7, Table 5), and the most economically-deprived will continue to be disproportionately represented amongst preventable unplanned hospital admissions [49].

### Economics of ADRe

Many ADRs and ADEs are preventable, and there can be significant resource and cost implications for health systems in failing to tackle the problem. Patients experiencing avoidable admissions and extended hospitalisation owing to avoidable ADR/ADE incur excess treatment costs of between €2,851 (£2,463) and €9,015 (£7,787) per patient [50]. The application of simple and inexpensive interventions such as ADRe within routine care has the potential to minimise preventable ADRs/ADEs. While the cost-effectiveness of any intervention must be demonstrated based on robust evidence of clinical efficacy and effectiveness, the median time required to complete ADRe was under 30 minutes, and in no cases exceeded an hour, suggesting that staff costs will be minimal. While it is difficult to accurately estimate wages for care home staff, a recently advertised post on www.carehome.co.uk, for a Care Home Clinical Lead Registered Nurse paid £17.50 per hour. With salary on-costs (in the UK, typically for pension contributions and employer national insurance contributions) of 15%, the cost per hour of labour is roughly £20.00 per hour. A crude calculation suggests that, at the lower bound of the cost of an ADR/ADE of about £2,400, one admission must be prevented for every 120 administrations of ADRe. Given that from 30 administrations, there were 17 referrrals, 5 interventions for falls, 1 for chest pain, and 6 for dyspnoea, it is likely that this threshold was achieved. This is a crude estimate based on the limited data available on both the costs of administering ADRe and the costs of ADRs for people living in care homes. However, it usefully illustrates that a low-tech intervention has the potential to provide benefit at health system level as well as patient level. The two other interventions identified in a systematic review of trials of medicines management interventions for people with dementia either depended on consultant input (increasing costs) or focused on medication adherence [51].

### From paper to electronic health records (EHRs)

**Fig 2:**
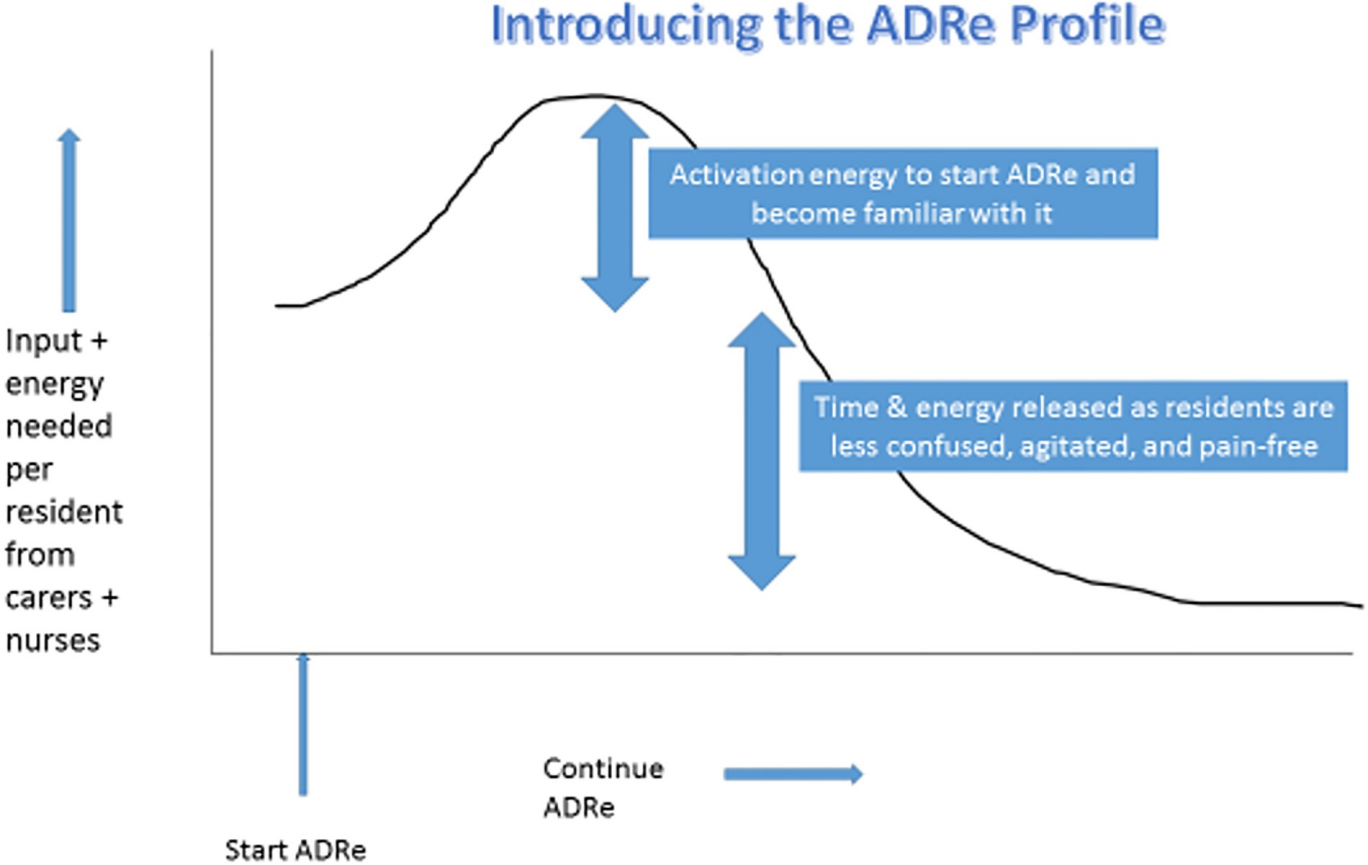
Energy and the ADRe profile. All things are difficult before they are easy, Thomas Fuller 1732. Fuller, T. (1732) *Gnomologia*: *Adages and Proverbs*, *Wise Sentences and Witty Sayings*, *Collected by Thomas Fuller*, No. 560, p.21. London: Barker/Bettesworth and Hitch. Available at: https://books.google.co.uk/books?id=3y8JAAAAQAAJ&printsec=frontcover#v=onepage&q&f=false.

Both time and EHRs were identified as barriers to implementation of ADRe, and these are inter-related. EHRs can identify severe events [19], but have increased NHS workloads [52], with electronic medicines optimisation a particular challenge [53]. Where clinical decision support is poor, electronic prescribing increases errors [54], and ADRe’s supporting information helps here. EHRs consume ~6 hours /day of responding doctors’ time and are associated with staff burnout in primary [55]and secondary care [56]. Electronic patient self-reporting of adverse events is effective in oncology [57] but most residents were too poorly to self-report. Although integration into EHRs overcomes some barriers (i.e. fitting into existing documentation, forcing responses to all items), it will not address others i.e. time, prescriber contact, staff turnover and overwhelming systems (Table 5). Introducing ADRe against this background of perceived stress and overwork is challenging. However, the initial time investment to complete ADRe in face-to-face interviews is repaid when residents feel they have been heard and are less confused and pain-free (Fig 2).

### ADRe focuses multidisciplinary team communication

**Fig 3:**
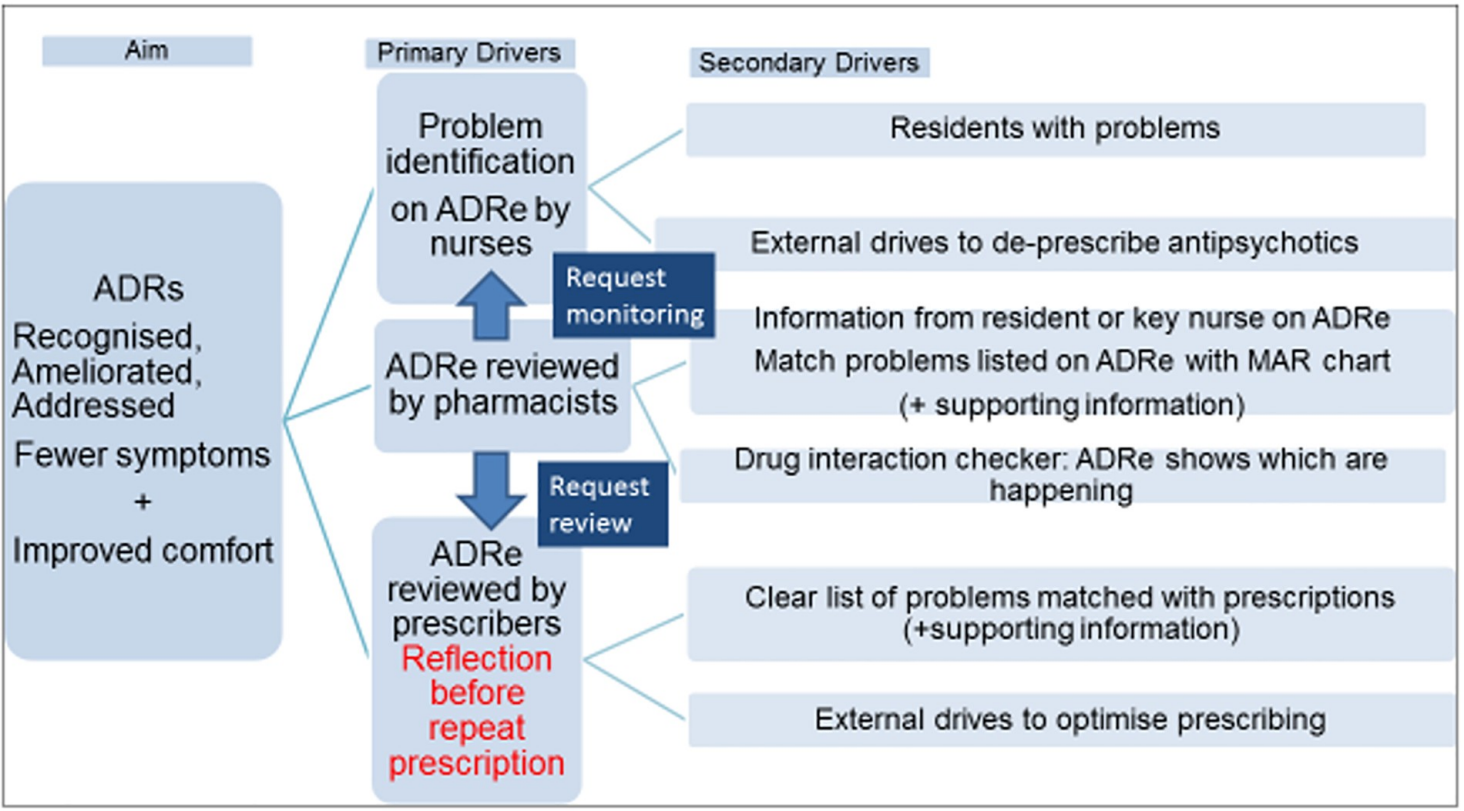
Integrating ADRe into multidisciplinary teams.

When medicines reviews are scheduled, giving pharmacists and prescribers a copy of ADRe alongside the MAR chart links residents’ problems to the clinical goal of freedom from ADRs (Fig 3) [58,59]. When reviewing medicines, pharmacists and doctors rarely have time to interview patients about every possible adverse effect of all their medicines, many residents are non-verbal, and the resident’s key carer is often off-duty, due to 12 hour shift patterns. For example, laxatives are commonly prescribed, but ADRe was needed to tell the pharmacist that the resident had diarrhoea, prompting adjusted prescribing (4 participants, Table 3). Some ~50% of ADRe’s signs and symptoms can be retrieved from patients’ notes, but this takes 1–1.5 hours [23, 24]. Medication reviews are labour-intensive [43], and under-resourced [60]; ADRe reduces overall costs by optimising pharmacists’ and prescribers’ time.

### Strengths and limitations

Linking problems with prescriptions *via* systematic nurse-led monitoring is a unique approach to a large problem that is threatening to overwhelm care delivery [21, 22], including hospital admissions [1–6]. Juxtaposition of prescriptions and problems clarified mechanisms for clinical improvements. Without this detail, the complexities of the cases would have been overlooked, problems would have remained unattended, and residents left in pain or falling. However, some ADRe items were omitted, particularly time-consuming vital signs, or unaddressed.

We acknowledge that this study is limited by selection bias, selection by prescriptions rather than diagnostic codes, size, and self-reporting in interviews. Although we focussed on objective clinical findings, no researchers can discount entrapment by prior expectation [61] or subconscious outcome reporting bias favouring benefits and discounting harm caused by the intervention. There is a risk that researcher expectancy bias will lead the study to exaggerate ADRe’s ability to detect problems and underplay implementation difficulties. However, we sought to ensure that problems identified were real by observation and using care homes’ own logs as evidence of adverse reactions or risks identified, and getting these verified by an independent pharmacist recruited to the team. We assessed ADRe’s “usability” from observations and staff reports in interviews, which were coded by multiple team members who were well aware that it would be counterproductive to exaggerate acceptability. Moreover, reporting was unaffected by the more pervasive and serious “reciprocation” or commercial interest biases [62].

Concerns over potential **volunteer bias** in care home selection emerged when 3 homes withdrew, citing difficulties in maintaining effective service delivery affecting their ability to participate: others note that only interested, “good” homes, with open-door policies welcome researchers [24, 63]. The observation that some of our homes were in economically deprived areas, with poor GP contact [48], increases the transferability of the findings, but we acknowledge generalisation of findings is based on logical, rather than statistical, inference [21] and can best be confirmed by replication studies of additional settings [64]. However, these findings show how residents *can* be helped, without sedation or exposure to risk from prescribed medicines: the challenges are to persuade everyone to emulate the best, to show that ADRe is easy to use and, with experience, the initial effort is more than repaid by clinical gain (Fig 2).

None of the 34 methods for **ADR causality assessment** is universally accepted [65] or easy to apply in practice [66]. This study identified putative ADRs, which were sometimes confirmed by de-prescribing. It was beyond the study’s scope to identify errors or to systematically de-challenge and re-challenge participants with suspected medicines to determine which, if any, of the prescribed medicines was responsible in each case. Re-challenge is rarely practical, and formal assessments of ADR causality cannot confer certainty, prove a connection or quantify a contribution [67]; they rarely achieve consensus [68].

The study’s limited duration, resources, and size precluded detailed follow up of all participants, and uncommon events. The 30 detailed cases indicated data saturation, and triangulation of stakeholders’ views with cases (Table 5) demonstrated how ADRe can and should work.

## Implications for practice: The case for change

ADRe is needed to minimise pain, dyspnoea, sedation, confusion, insomnia, EPS, falls, incontinence, and aggression. The problems addressed here permeate health and social care services [2, 8, 12]. The change in thinking to reflectively link problems with known ADRs is a barrier to be negotiated, but the pervasiveness of unmonitored harm and entrapment by prior expectation are profound challenges [2, 8, 12, 69]. Adjusting healthcare systems to check systematically for iatrogenic harm may be difficult until the benefits of minimising medicines-related harm are understood, the process is normalised [70] and gatekeepers accept that patients need strategies to communicate their signs, symptoms and concerns [71].

Demographic and prescribing changes are increasing the need for a comprehensive, systematic multi-professional approach to medicines optimisation. In the UK in 2008–11, 49% (of 7359) of people aged 65 and over took more than four medicines, a fourfold increase since 1991–5 [72], whilst emergency admissions for ADRs rose [73]. Life expectancy is no longer rising [74].

The WHO asks health ministries to programme changes in professionals’ behaviour, systems and practices of medication management to reduce ADRs, polypharmacy and miscommunication [75, 76]. Workforce constraints suggest this may be best achieved by extending and expanding nurses’ roles, building on nurses’ knowledge and communication skills. ADRe can do this and address long-standing problems. Presenting evidence to gatekeepers is insufficient [77] change is mediated through social networks, power structures, and professional politics. The challenge is to persuade policy makers that a person-centred [78], clinically and cost-effective tool, such as ADRe, [24] deserves support.

Note: In the Welsh language “Adre” means homewards. We hope the ADRe profile will bring a sense of greater security for those prescribed mental health medicines.

## Supporting information

S1 File Revised Standards for Quality Improvement Reporting Excellence SQUIRE2.0 plus Consolidated criteria for reporting qualitative studies (COREQ). (DOCX)

S2 File Interview Schedule. (DOCX)

S3 File Supplementary Tables. (DOCX)
