# Supplementary material for: Nurse-led medicines’ monitoring in care homes, implementing the Adverse Drug Reaction (ADRe) Profile improvement initiative for mental health medicines: An observational and interview study
Source: PLoS One. 2019 Sep 11;14(9):e0220885. doi: 10.1371/journal.pone.0220885 (PMC6738583; doi:10.1371/journal.pone.0220885)
Supplement: S1 File — (DOCX) [file pone.0220885.s001.docx]

S1 File. Revised Standards for Quality Improvement Reporting Excellence 

SQUIRE 2.0 (COREQ is below)

| Notes to Authors | |  |
| --- | --- | --- |
| - The SQUIRE guidelines provide a framework for reporting new knowledge about how to improve healthcare. - The SQUIRE guidelines are intended for reports that describe [system](http://www.squire-statement.org/index.cfm?fuseaction=page.viewpage&pageid=485#System) level work to improve the quality, safety, and value of healthcare, and used methods to establish that observed outcomes were due to the [intervention(s).](http://www.squire-statement.org/index.cfm?fuseaction=page.viewpage&pageid=485#Interventions) - A range of approaches exists for improving healthcare.  SQUIRE may be adapted for reporting any of these. - Authors should consider every SQUIRE item, but it may be inappropriate or unnecessary to include every SQUIRE element in a particular manuscript. - The SQUIRE Glossary contains definitions of many of the key words in SQUIRE. - The [Explanation and Elaboration](http://www.squire-statement.org/index.cfm?fuseaction=page.viewpage&pageid=504)document provides specific examples of well-written SQUIRE items, and an in-depth explanation of each item. - Please cite SQUIRE when it is used to write a manuscript. | |  |
| Title and Abstract | |  |
| **1.  Title** | Indicate that the manuscript concerns an [initiative](http://www.squire-statement.org/index.cfm?fuseaction=page.viewpage&pageid=485#Initiative) to improve healthcare (broadly defined to include the quality, safety, effectiveness, patient-centeredness, timeliness, cost, efficiency, and equity of healthcare)  **Nurse-led medicines’ monitoring in care homes, implementing the Adverse Drug Reaction (ADRe) Profile improvement initiative for Mental Health Medicines: an observational and interview study** |  |
| **2.  Abstract** | a.  Provide adequate information to aid in searching and indexing  b.  Summarize all key information from various sections of the text using the abstract format of the intended publication or a structured summary such as: background, local [problem](http://www.squire-statement.org/index.cfm?fuseaction=page.viewpage&pageid=485#Problem), methods, interventions, results, conclusions  we have used ‘implications’ rather than conclusions |  |
| Introduction | *Why did you start?* |  |
| [**3. Problem Description**](http://www.squire-statement.org/index.cfm?fuseaction=page.viewpage&pageid=485#Problem) | Nature and significance of the local [problem](http://www.squire-statement.org/index.cfm?fuseaction=page.viewpage&pageid=485#Problem)  **Page 1 lines 2 - 17** |  |
| **4. Available Knowledge** | Summary of what is currently known about the [problem](http://www.squire-statement.org/index.cfm?fuseaction=page.viewpage&pageid=485#Problem), including relevant previous studies  **Page 1 lines 2-17** |  |
| **5. Rationale** | Informal or formal frameworks, models, concepts, and/or [theories](http://www.squire-statement.org/index.cfm?fuseaction=page.viewpage&pageid=485#Theory) used to explain the [problem](http://www.squire-statement.org/index.cfm?fuseaction=page.viewpage&pageid=485#Problem), any reasons or [assumptions](http://squire.citysoft.org/index.cfm?fuseaction=page.viewPage&pageID=485&nodeID=1#assumptions) that were used to develop the [intervention(s),](http://www.squire-statement.org/index.cfm?fuseaction=page.viewpage&pageid=485#Interventions)and reasons why the [intervention(s)](http://www.squire-statement.org/index.cfm?fuseaction=page.viewpage&pageid=485#Interventions) was expected to work  **Page 1**  **Why the intervention is expected to work: lines 19-24**  **Why this intervention chosen : lines 13-17** |  |
| **6. Specific Aims** | Purpose of the project and of this report  **Page 1 lines 28-31** |  |
| Methods | *What did you do?* |  |
| [**7. Context**](http://www.squire-statement.org/index.cfm?fuseaction=page.viewpage&pageid=485#context) | Contextual elements considered important at the outset of introducing the [intervention(s)](http://www.squire-statement.org/index.cfm?fuseaction=page.viewpage&pageid=485#Interventions)  **P 2**  **Recruitment and collaboration - lines 36-40**  **Physical environment or Setting – lines 42 -45**  **Sense – making/interpretation of the environment and intervention: lines 44 – 45; 47-51** |  |
| [**8. Intervention(s)**](http://www.squire-statement.org/index.cfm?fuseaction=page.viewpage&pageid=485#Interventions) | a.  Description of the [intervention(s)](http://www.squire-statement.org/index.cfm?fuseaction=page.viewpage&pageid=485#Interventions) in sufficient detail that others could reproduce it  **P 2 lines 33-34 refers to study protocol for further information about the intervention ADRe (See reference 21).**  **P3 Description of observations of implementation of intervention (ADRe) lines 65-68**  **Page 33 Fig 3 logic model** |  |
| **9. Study of the Intervention(s)** | **a**.  Approach chosen for assessing the impact of the [intervention(s)](http://www.squire-statement.org/index.cfm?fuseaction=page.viewpage&pageid=485#Interventions)  **p.2 lines 54-57**  **b.  Approach used to establish whether the observed outcomes were due to the**[**intervention(s)**](http://www.squire-statement.org/index.cfm?fuseaction=page.viewpage&pageid=485#Interventions)  **p2 line 49-50** |  |
| **10. Measures** | a.  Measures chosen for studying [processes](http://www.squire-statement.org/index.cfm?fuseaction=page.viewpage&pageid=485#Process) and outcomes of the [intervention(s),](http://www.squire-statement.org/index.cfm?fuseaction=page.viewpage&pageid=485#Interventions)including rationale for choosing them, their operational definitions, and their validity and reliability  b.  Description of the approach to the ongoing assessment of contextual elements that contributed to the success, failure, efficiency, and cost  c.  Methods employed for assessing completeness and accuracy of data  **a. page 2 line 53-60**  **b. p.2 lines 58-60**  **c. p. 3 lines 81-82 see protocol paper reference 21for further details** |  |
| **11. Analysis** | a.  Qualitative and quantitative methods used to draw [inferences](http://www.squire-statement.org/index.cfm?fuseaction=page.viewpage&pageid=485#Inferences) from the data  **p.3 observations 65-68**  **p.3 interviews lines 70 – 78**  **Analysis**  **Page 3 80-82; p16 lines 170-171**  **Methods for understanding variation within the data**  **p.3 lines 81-82** **See protocol paper ref 21** |  |
| **12. Ethical Considerations** | [Ethical aspects](http://www.squire-statement.org/index.cfm?fuseaction=page.viewpage&pageid=485#Ethical_aspects)of implementing and studying the [intervention(s)](http://www.squire-statement.org/index.cfm?fuseaction=page.viewpage&pageid=485#Interventions) and how they were addressed, including, but not limited to, formal ethics review and potential conflict(s) of interest  **P3 84-89** |  |
| Results | *What did you find?* |  |
| **13. Results** | a.  Initial steps of the [intervention(s)](http://www.squire-statement.org/index.cfm?fuseaction=page.viewpage&pageid=485#Interventions) and their evolution over time (e.g., time-line diagram, flow chart, or table), including modifications made to the intervention during the project  **P 4 Recruitment and modifications to recruitment – lines 92 – 103; See Fig 1 page 32 lines 487 -511**  b.  Details of the [process](http://www.squire-statement.org/index.cfm?fuseaction=page.viewpage&pageid=485#Process) measures and outcome  **Summary of outcomes page 5 Table 2**  **Clinical Impact page 5 lines 129-137; tables S1a, S1b**  **Clinical gains Table 4 pages 12 – 15**  **Potential gains identified by pharmacist Table2, 3 p 6 & 7**  c.  Contextual elements that interacted with the [intervention(s)](http://www.squire-statement.org/index.cfm?fuseaction=page.viewpage&pageid=485#Interventions)  **p.4 lines 107 - 119**  d.  Observed associations between outcomes, interventions, and relevant contextual elements   **Page 6 lines 139- 147 See table3 page 7-10**  e.  Unintended consequences such as unexpected benefits, [problems](http://www.squire-statement.org/index.cfm?fuseaction=page.viewpage&pageid=485#Problem), failures, or costs associated with the [intervention(s).](http://www.squire-statement.org/index.cfm?fuseaction=page.viewpage&pageid=485#Interventions)  **Problems - P 4 lines 113-119; P 11 157-163;**  f.  Details about missing data  **Limitations p 27 lines 379- 580** |  |
| Discussion | *What does it mean?* |  |
| **14. Summary** | a.  Key findings, including relevance to the [rationale](http://www.squire-statement.org/index.cfm?fuseaction=page.viewpage&pageid=485#Rationale) and specific aims  **Key findings - p 27 lines 365 – 373; linking to rationale p27 lines 375-376; linking to aims p28 lines 399-400**  b.  Particular strengths and weaknesses of the project  **p. 27 –lines 374 - 400** |  |
| **15. Interpretation** | 1. Nature of the association between the [intervention(s)](http://www.squire-statement.org/index.cfm?fuseaction=page.viewpage&pageid=485#Interventions) and the outcomes   **table 3 fig 3 Discussion of causality page 28 lines 390- 396**   1. Comparison of results with findings from other publications   **P 28 402 -450; 462 - 440**   1. Costs and strategic trade-offs, including [opportunity costs](http://www.squire-statement.org/index.cfm?fuseaction=page.viewpage&pageid=485#Opportunity_costs)   **Page 29 Discussion of financial costs and implications lines 420-440** |  |
| **16. Limitations** | a.  Limits to the [generalizability](http://www.squire-statement.org/index.cfm?fuseaction=page.viewpage&pageid=485#Generalizability) of the work  **P 28 lines 381-382**  b.  Factors that might have limited [internal validity](http://www.squire-statement.org/index.cfm?fuseaction=page.viewpage&pageid=485#Internal_validity) such as confounding, bias, or imprecision in the design, methods, measurement, or analysis  **Discussion of volunteer bias – page 28 lines 383 - 385**  c.  Efforts made to minimize and adjust for limitations  **discussion p 27- 28 strengths and limitations** |  |
| **17. Conclusions** | 1. Usefulness of the work   **page 28 lines 386-389 and fig 2**   1. Sustainability   **Table 5**  c.  Potential for spread to other [contexts](http://www.squire-statement.org/index.cfm?fuseaction=page.viewpage&pageid=485#context)  **p 29 lines 441 - 450**   1. Implications for practice and for further study in the field -   **p 30 lines 459- 460; 462 - 478**  e.  Suggested next steps  **p.30 lines 477 - 488** |  |
| Other Information |  |  |
| **18. Funding** | Sources of funding that supported this work. Role, if any, of the funding organization in the design, implementation, interpretation, and reporting  Statement p.31 |  |

COREQ guidelines

## Nurse-led medicines’ monitoring in care homes, implementing the Adverse Drug Reaction (ADRe) Profile improvement initiative for Mental Health Medicines: an observational and interview study

This is an observation/ interview study evaluating an improvement initiative, with both qualitative and quantitative data. We have also completed the SQUIRE template for Quality Improvement Reporting Excellence, as this more closely reflects the study methods. Many of the methods questions are addressed fully in the published, open access, protocol paper (Jordan et al 2018).

Jordan S, Banner T, Gabe-Walters M the Medicines Management Group*, et al* Nurse-led medicines’ monitoring in care homes study protocol: a process evaluation of the impact and sustainability of the adverse drug reaction (ADRe) profile for mental health medicines *BMJ Open*2018;**8:**e023377. doi: 10.1136/bmjopen-2018-023377 <http://bmjopen.bmj.com/cgi/content/full/bmjopen-2018-023377>

**Table 1**

Consolidated criteria for reporting qualitative studies (COREQ): 32-item checklist

| **No** | **Item** | **Guide questions/description** | **Response** | **page** |  |
| --- | --- | --- | --- | --- | --- |
| **Domain 1: Research team and reflexivity** |  |  |  |  |  |
| Personal Characteristics |  |  |  |  |  |
| 1. | Interviewer/facilitator | Which author/s conducted the interview or focus group? | SJ, MGW, DH, JM, SS, MS  This was an observation and interview study. | 3 |  |
| 2. | Credentials | What were the researcher's credentials? *E.g. PhD, MD* | Only MS does not hold a PhD. She has extensive experience of interview studies |  |  |
| 3. | Occupation | What was their occupation at the time of the study? | SJ, SS & DH are academics. MS, JM and MGW were research assistants |  |  |
| 4. | Gender | Was the researcher male or female? | Only DH is male. Most care home staff are female. | 3 |  |
| 5. | Experience and training | What experience or training did the researcher have? | All are experienced researchers, except JM, who observed DH interviewing on several occasions. | 3 |  |
| Relationship with participants |  |  |  |  |  |
| 6. | Relationship established | Was a relationship established prior to study commencement? | 5 care homes had worked on a previous trial. 5 were newly recruited. | 3 |  |
| 7. | Participant knowledge of the interviewer | What did the participants know about the researcher? e*.g. personal goals, reasons for doing the research* | As above, they had seen the earlier papers. | 3 |  |
| 8. | Interviewer characteristics | What characteristics were reported about the interviewer/facilitator? e.g. *Bias, assumptions, reasons and interests in the research topic* | Authorship of the previous papers is transparent and public domain. | 28 |  |
| **Domain 2: study design** |  |  |  |  |  |
| Theoretical framework |  |  |  |  |  |
| 9. | Methodological orientation and Theory | What methodological orientation was stated to underpin the study? *e.g. grounded theory, discourse analysis, ethnography, phenomenology, content analysis* | A grounded theory approach was taken to interview analysis.  The observation template is in tables S1a & b | 4 |  |
| Participant selection |  |  |  |  |  |
| 10. | Sampling | How were participants selected? *e.g. purposive, convenience, consecutive, snowball* | 5 care homes had participated in the previous trial. The new homes were consecutively recruited. | 4 |  |
| 11. | Method of approach | How were participants approached? e*.g. face-to-face, telephone, mail, email* | Email, following a presentation. | 2 |  |
| 12. | Sample size | How many participants were in the study? | 30 interviews + 30 observations. | 3 |  |
| 13. | Non-participation | How many people refused to participate or dropped out? Reasons? | 3 care homes. 1 resident. 1 home closed, 2 found ADRe too difficult. Resident’s reason not reported by nurse. We cannot disclose further information. | 5 |  |
| Setting |  |  |  |  |  |
| 14. | Setting of data collection | Where was the data collected? e*.g. home, clinic, workplace* | Observations in care homes. Interviews in care homes, own homes, offices and cafes. | 3 and 4 |  |
| 15. | Presence of non-participants | Was anyone else present besides the participants and researchers? | Some interviews were observed by researchers. Some service users had a partner present. | 3 |  |
| 16. | Description of sample | What are the important characteristics of the sample? *e.g. demographic data, date* | Care home stakeholders  Residents. | 2  2  5 |  |
| Data collection |  |  |  |  |  |
| 17. | Interview guide | Were questions, prompts, guides provided by the authors? Was it pilot tested? | Yes. Yes, in previous work (Jordan et al 2000, 2002), and as referenced. | 3  Interview guide is in supplementary material. |  |
| 18. | Repeat interviews | Were repeat interviews carried out? If yes, how many? | None |  |  |
| 19. | Audio/visual recording | Did the research use audio or visual recording to collect the data? | Audio | 4 |  |
| 20. | Field notes | Were field notes made during and/or after the interview or focus group? | Yes | 3 |  |
| 21. | Duration | What was the duration of the interviews or focus group? | From 20 to 60 minutes. Service user and government officials gave shorter interviews |  |  |
| 22. | Data saturation | Was data saturation discussed? | Yes | 4 |  |
| 23. | Transcripts returned | Were transcripts returned to participants for comment and/or correction? | No. This would have increased the respondent burden, and many respondents were under time pressures or had poor recollection. We aimed to capture the immediate issues. |  |  |
| **Domain 3: analysis and findings**z |  |  |  |  |  |
| Data analysis |  |  |  |  |  |
| 24. | Number of data coders | How many data coders coded the data? | 4. SJ, DH, SS, MS | 4 |  |
| 25. | Description of the coding tree | Did authors provide a description of the coding tree? | Yes, table 5, S3 | Table 5 p.16 |  |
| 26. | Derivation of themes | Were themes identified in advance or derived from the data? | The main themes were derived from previous work, as in the protocol. The subthemes emerged from the data. | 4 |  |
| 27. | Software | What software, if applicable, was used to manage the data? | None |  |  |
| 28. | Participant checking | Did participants provide feedback on the findings? | Yes, nurses feedback on the observations at interview. | 2,4 |  |
| Reporting |  |  |  |  |  |
| 29. | Quotations presented | Were participant quotations presented to illustrate the themes / findings? Was each quotation identified? e*.g. participant number* | Yes  Yes, throughout | 12-15  16-20  24-27 |  |
| 30. | Data and findings consistent | Was there consistency between the data presented and the findings? | Yes | Throughout. Tables 4 & 5 illustrate this. |  |
| 31. | Clarity of major themes | Were major themes clearly presented in the findings? | Yes, subheadings 1-3 of results section. | Tables 4 & 5 are built around themes. |  |
| 32. | Clarity of minor themes | Is there a description of diverse cases or discussion of minor themes? | Cases are presented in tables 3-5 and S2 | Table 3 – p7  Table 4 – p.11  Table 5 – p.16 |  |

[View Large](https://academic.oup.com/view-large/27217733)

References

- Jordan S. 2002 Managing Adverse Drug Reactions: An Orphan Task. Developing Nurse-Administered Evaluation Checklists *Journal of Advanced Nursing* ; 38 : 5 : 437-48

# Jordan S., Tunnicliffe C., Sykes A. 2002 Minimising Side Effects: The clinical impact of nurse-administered ‘side effect’ checklists. *Journal of Advanced Nursing*. 37; 2 : 155-65

- Jordan S., Philpin S., Davies S., Andrade M. 2000 The biological sciences in mental health nursing: stakeholders’ perspectives. *Journal of Advanced Nursing* : 32 : 4 : 881-91
