# Supplementary material for: Nurse-led medicines’ monitoring in care homes, implementing the Adverse Drug Reaction (ADRe) Profile improvement initiative for mental health medicines: An observational and interview study
Source: PLoS One. 2019 Sep 11;14(9):e0220885. doi: 10.1371/journal.pone.0220885 (PMC6738583; doi:10.1371/journal.pone.0220885)
Supplement: S2 File — (DOCX) [file pone.0220885.s002.docx]

## S2 File. Interview Schedule and Topic Guide

## Nurse-led medicines’ monitoring in care homes: implementation of the Adverse Drug Reaction (ADRe) Profile improvement initiative for Mental Health Medicines an observational and interview study

The content was adjusted for each respondent group.

#### Interviews aim to understand:

- Clinical gain (anticipated and actual)
- Barriers and facilitators
- Change needed

**Experience, expectations and views of the ‘side effects’ of the drugs prescribed for mental health.**

What is your experience of patient’s/ service users’ medication management?

What side effects have you observed in your patients taking medication for mental health problems?

What would you expect health care professionals to do to minimise side effects?

What actions are in place to minimise such side effects developing?

Do you have any thoughts on anything further that could be done to minimise such side effects?

What measures have you put in place here to monitor your patients’ medications?

**Prioritisation of / how important is medication monitoring?**

- On a scale of 1 – 10 with 10 being very important and 1 not that important Where would you place medicine monitoring?

**Communications between prescribers, pharmacists, nurses and patients**

What are your views on communication between doctors prescribing, nurses administrating and pharmacists dispensing?

What prevents or complicates this communication?

**What are your views on the use of the Profile & its guidelines?**

- If relevant: Who has benefited from the Profile? Why did s/he benefit?
- How can we avoid this being a paper / bureaucratic exercise?
- Does anything need to change before Profiles can be implemented regularly?
- What are the barriers to implementation / in your home / in the inspectorate’s criteria/ in policy documentation?
- How can we capture / audit the impact of medication monitoring? E.g. dispensed items?
- Would Profiles for other medicines (e.g. diuretics – show example) be useful?
- What should we do now to implement?
