# Supplementary material for: Nurse-led medicines’ monitoring in care homes, implementing the Adverse Drug Reaction (ADRe) Profile improvement initiative for mental health medicines: An observational and interview study
Source: PLoS One. 2019 Sep 11;14(9):e0220885. doi: 10.1371/journal.pone.0220885 (PMC6738583; doi:10.1371/journal.pone.0220885)
Supplement: S3 File — (DOCX) [file pone.0220885.s003.docx]

## S3 File: Supplementary Tables

### Table A1. Profile items and responses: vital signs

| **Item on ADRe** | **Problem** | | **Other** | **Missing** | **Description / overview of problems noted on ADRe** | **Number actioned*** | **Examples of Actions Recorded** |
| --- | --- | --- | --- | --- | --- | --- | --- |
|  | **No** | **Yes** |  |  |  |  |  |
| Heart Rate Problem recorded | 25 | 1 |  | 4 | Machine fault | 0 |  |
| HR within normal limits (50-90 bpm) | 1 | 23 |  | 6 | 91 bpm | 0 |  |
| Irregular Heart Rhythm | 27 | 0 |  | 3 |  | NA |  |
| BP Lying or sitting Problem marked | 24 | 2 |  | 7 | 6 had no recordings. For 1 resident with SBP <110mmHg the question was left blank. SBPs of 103 & 105mmHg were marked as ‘no problem’. | 2 | Rechecking for 2 cases of hypertension by care staff |
| BP within normal limits (SBP 111-140 mmHg) | 8 | 16 |  | 6 | 3 <110mm Hg, 1 >170mmHg, 4 140-160mmHg | 2 | Rechecking for 2 cases of hypertension by care staff |
| BP Standing Problem marked | 16 | 3 |  | 11 | 3 residents noted unable to stand, 2 devices noted as faulty, 6 marked as ‘no problem’ when standing BP had not been taken. | 3 | Rechecking, falls risk assessment, advice to stand slowly. |
| Postural Hypotension y/n (SBP difference >19mmHg) | 9 | 2 | 2 borderline | 17 | postural drops 36, 32, 17 & 14mmHg | 3 | Rechecking, falls risk assessment, advice to stand slowly. |
| Weight BMI Problem | 19 | 6 | 2 worsening | 3 | weight up to 145 kg | 2 | mid arm circumference checked, contacted prescriber |
| Weight change | 12 | 12 |  | 6 | 4 gain, 8 loss (highest 2.3 kg after inpatient stay)  Some residents were noted as uncooperative. | 11 | Diet diary in 8 cases, e.g. to identify snacks. Finger feeding offered to address weight loss. |
| Girth Problem indicated | 8 | 2 |  | 20 | Some residents too immobile to be measured. | 1 | Diet diary |
| Girth within normal limits <88 or <102 cms) | 8 | 3 | 1 borderline | 18 | Girths up to 140 cm were recorded. Nurses noted that 1 resident was shaking too much and it was impossible to get the tape round others to allow accurate readings. | 1 | Diet diary |
| Temperature Problem | 17 | 2 |  | 11 |  | O |  |
| Temperature within normal limits (36.1 - 38.0⁰C) | 2 | 10 | 2 borderline | 16 | 35.4 and 35.9⁰C, likely incomplete contact | 0 |  |
| Oxygen Saturation Problem marked | 23 | 3 |  | 4 |  | 3 | Rechecking, but no action if still low |
| Oxygen Saturation within normal limits (>96%) | 10 | 15 |  | 5 | lowest 89% | 3 | Residents noted as smokers or asthmatic. Rechecking. |
| ECG available | 30 | 0 |  | 30 | not available | 0 | Nurses were aware of the situation |

### Table A2. Profile items and responses: observations and questions

| **Item on ADRe** | **Problem** | | **Missing** | **Description of problem on ADRe** | **Number actioned*** | **Examples of actions recorded** |
| --- | --- | --- | --- | --- | --- | --- |
|  | **No** | **Yes** |  |  |  |  |
| Hand Tremor | 21 | 9 | 0 | L sided wobble, "always been there" | 0 |  |
| Tongue Tremor | 25 | 5 | 0 | None | 0 |  |
| Feet Shuffling | 18 | 12 | 0 | None | 0 |  |
| Abnormal Movement Resting | 23 | 6 | 1 | restless leg syndrome | 0 |  |
| Posture Abnormal | 20 | 9 | 1 | stooped, bent over | 0 |  |
| Gait Abnormal | 19 | 8 | 3 | Shuffles | 0 |  |
| Balance | 16 | 14 | 0 | attributed to dementia | 1 | watched all the time |
| Bleeding | 28 | 2 | 0 | rash on legs | 0 |  |
| Feeling cold | 15 | 14 | 1 | "wears hat and fleece indoors" | 1 | Given own blankets at night |
| Cognitive Decline | 8 | 21 | 0 | dementia, depression, fluctuating | 0 |  |
| CNS - convulsions | 20 | 7 | 3 | Epileptic | 0 |  |
| Behavioural Problems | 14 | 15 | 1 | self-neglect, swearing, aggressive, restless agitated | 0 |  |
| Self-harm | 27 | 3 | 0 | picking skin | 0 |  |
| Physical Violence | 24 | 6 | 0 | Rare | 0 |  |
| Aggression | 19 | 11 | 0 | shouts, argues, dementia, nightly | 0 |  |
| Irritability | 13 | 17 | 0 | argues, at night | 0 |  |
| Agitation | 8 | 22 | 0 | nervousness, co-residents, anxiety, "about getting old" | 0 |  |
| Restlessness | 15 | 15 | 0 | at night, picks on others | 0 |  |
| Hyperactivity | 24 | 5 | 1 | at night, after alcohol | 0 |  |
| Panic Attacks | 28 | 2 | 0 | Sometimes | 0 |  |
| Confusion | 8 | 22 | 0 | dementia, "shouts for late wife", relives bereavement | 0 |  |
| Mood Fluctuations | 13 | 17 | 0 | up & down, mainly high, no triggers | 0 |  |
| Low Energy | 16 | 13 | 1 | depressed, restless, not sleeping | 0 |  |
| Hallucinations | 22 | 7 | 1 | "better since haloperidol reduced", voices, "seeing people", "sees late wife", nightmares since husband fell off roof, unable to speak | 0 |  |
| Sleep Problems | 15 | 14 | 1 | looks for food, sleeps in the day, pain, TV at night, sleeping tablets | 2 | monitor, door sensor |
| Sedation | 24 | 5 | 1 | restless at night | 1 | Risperidone now PRN |
| Dizziness | 22 | 7 | 1 | on standing, after UTI & pneumonia, ?eyesight | 1 | refer to registered nurse to prevent falls |
| Falls | 20 | 9 | 1 | blackouts, before admission | 5 | risk assessment, care planning |
| Headaches | 18 | 10 | 2 | sometimes, history of migraines | 2 | "look out for signs", Paramax® |
| Tinnitus | 26 | 2 | 2 | concentration problems | 0 |  |
| Tingling | 24 | 4 | 2 | problems in arms and fingers | 0 |  |
| Urination problem / incontinence | 14 | 16 | 0 | catheter, pads, UTI | 1 | Monitoring |
| Burning urine / UTI | 25 | 4 | 1 | Thrush, cystitis whilst in hospital | 0 |  |
| Problems with reproductive system | 22 | 3 | 5 | Enlarged prostate, discomfort L breast, masturbates. Marked as too sensitive to ask, twice. | 0 |  |
| Chest Pain | 28 | 2 | 0 | ? Due to inhalers, previous pneumonia | 1 | GP contacted |
| Short of Breath | 24 | 6 | 0 | "doesn't talk much" | 6 | All referred to prescribers |
| Hyper-salivation | 26 | 4 | 0 | "a little", "all the time" | 0 |  |
| Nausea/ Vomiting | 29 | 1 | 0 | Colostomy | 0 |  |
| Appetite / Taste | 26 | 4 | 0 | diabetic diet | 1 | smaller portions |
| Bowel Control | 24 | 5 | 1 | constipated - on iron | 1 | further checks |
| Constipation | 22 | 7 | 1 | already taking 2 laxatives | 1 | Review of iron prescription |
| Skin Rash | 25 | 5 | 0 | dry skin, eczema, itching | 0 |  |
| Swelling | 26 | 4 | 0 | "taking furosemide" | 1 | checked Waterlow score |
| Sweating | 27 | 2 | 1 | "only head" | 0 |  |
| Sore Throat | 28 | 2 | 0 | "for a while" | 1 | checked temperature (within normal limits) |
| Injection Site Problems | 30 | 0 | 0 |  | NA |  |
|  |  |  |  |  |  |  |
| Intake: misses meals or leaves unfinished more than once / day | 25 | 5 | 0 | "over-eats, no control", loves food, | 10 | diet diary, reminders to eat |
| Snacking | 8 | 21 | 1 | eats chocolate daily, elevenses + afternoon snack | 2 | encouraged to eat, forgets he has eaten |
| Milk Intake: at least 1 pint per day. | 6 | 23 | 1 | cereal with orange juice, When awake | 0 |  |
| Vitamin D deficiency risk e.g. no sunlight exposure | 6 | 20 | 4 | check diet with carers | 4 | fish every Friday, sit out, check for prescription |
| Fruit/Veg Intake daily | 3 | 25 | 2 | dislikes vegetables, refuses fruit | 5 | list intake to check |
| Drinking 6-8 cups/ day | 1 | 28 | 1 | drinks plenty, "when awake" | 1 | "needs to drink more" |
| Drinks Sugar Free | 8 | 20 | 2 | sugar in tea | 0 |  |
| Swallowing difficulties | 23 | 5 | 2 | stores food in her mouth, choses purees | 1 | SALT assessment |
| Indigestion /Heartburn | 25 | 4 | 1 | with cheese and chocolate | 1 | treating with Adcal d3 |
| Dental Problem | 25 | 5 | 0 | "Doesn't brush his teeth", loose, due to weight loss" | 0 |  |
| Dry Mouth | 25 | 5 | 0 | "due to oxygen" | 0 |  |
| Halitosis | 27 | 3 | 0 |  | 1 | dentist said there was no problem |
| Dental Visit in last year | 5 | 23 | 2 | bad teeth, there was an appointment 6 months ago, can't remember | 1 | due in 2 weeks |
| Smoking | 29 | 1 | 0 | 10/ day | 0 |  |
| Changes in Smoking | 28 | 1 | 1 | change dated | 0 |  |
| Vision Problems | 18 | 11 | 1 | loses/ refuses glasses, TV/ reading a problem | 3 | arranged treatment for red eye |
| Optician Visit in last year | 1 | 29 | 0 |  | 1 | waiting to see optician |
| Sun Screen available | 2 | 26 | 2 | stays indoors | 0 |  |
| Glasses for bright sunlight | 22 | 7 | 1 | wears a sunhat, does not go out | 0 |  |
| Hair Loss | 24 | 6 | 0 | Thinning | 0 |  |
| Acne / herpes simplex | 27 | 3 | 0 | in the past | 0 |  |
| Pain | 22 | 7 | 1 | location stated | 6 | paracetamol / co-dydramol given |
| Non Verbal Pain Checked | 3 | 26 | 1 | asked, look, grimaces | 1 | 1 additional resident given analgesia. Pain already actioned for others based on question above |
| Medicines administered regularly | 4 | 26 | 0 | [Administered] if required | 1 | think of errors |
| Tablets Crushed | 29 | 1 | 0 |  | 0 |  |
| Doses missed | 26 | 4 | 0 | if sleeping, only if required | 0 |  |
| Non-prescription medicines used | 29 | 1 | 0 | Cologne for a headache | 0 |  |
| Recreational drug use | 30 | 0 | 0 |  | 0 |  |
| Alcohol Use | 28 | 2 | 0 | Drunk after last visit to relatives | 0 |  |
| Any Other Problems | 26 | 2 | 2 | Pain, waking at 2.00 am, 2 residents gave no response; a further 4 were noted as unable to speak. | 0 |  |
| Health Problems important to you | 18 | 6 | 6 | Dental problems x2, something wrong with my heart, incontinence, pain, unable to breathe, forgetful, breast cancer, cough. 6 residents gave no response; 4 others were noted as unable to speak. | 0 |  |
| Message for Prescriber | 20 | 5 | 5 | This research is a good thing, Do your job - see me when asked, Help with blackouts, pain, and medicines review. 5 residents gave no response, 4 others were unable to speak. | 0 |  |
| Optional Diet Diary Done | 18 | 11 | 1 | For a woman at risk of exceeding the weight limit on the hoist: ice-cream, trifle, cake, biscuits, cheese & crackers, crisps in addition to breakfast, beef dinner and sandwiches | 6 | Kept under review. Not all diaries were completed in response to weight problem or change. |

*Other actions were added to care plans or narrated to researchers in the debrief interviews.

### TableB. Clinical gains, barriers and facilitators: triangulation of observations, profiles and interviews with case examples

| **Participant** | **Theme** | **Case Reports** | **Problems identified on ADRe** | **Extracts from Interviews** |
| --- | --- | --- | --- | --- |
|  | **Clinical gains** | (see table 2) |  | **Mechanism / How it happened** |
| 3.2  Woman, 84 | Patient more contented following reduction of antipsychotics. | When ADRe was shared with the consultant, haloperidol was reduced, then stopped. PRN was retained, but rarely used. *Mood settled, and 3.2 said she felt safe at night, and slept at ease. Underactive thyroid identified and treated. P*rescriptions:  1.folic acid 5 mg od  2. clopidogrel 75mg od  3. Isosorbide mononitrate 60mg. half tablet  4. amlodipine 10mg od  5.atorvastatin 20mg od- night  6. ramipril 2.5mg  od- night  7.sertraline 50mg. od  8.adcal-d3 chewable tutti frutti.bd  9. clarithromycin 250mg bd from 16.2-5.3.16  10. paracetamol 500mg 2 every 4-6 hrs 4 x day  11. senna 7.5mg - 2- nocte  12. haloperidol 0.5mg bd- morn & night  12. ropinirole 0.5mg bd  13. risedronate 35mg once/week same day | 1. Weight increasing 2. borderline oxygen, 3. hallucinations, 4. weight gain, 5. tremor, 6. tongue tremor, 7. shuffling, 8. abnormal movements, 9. posture, 10. feels cold, 11. irritable, 12. restless, 13. incontinence, 14. sob, 15. snacking, 16. hair loss   diet diary indicated cakes and snacks. | We look at the daily logs and the profile when we do their monthly care plans to see if we can find a reason. (…) So when the consultant team come, we can present the case. Obviously, the doctors are only coming and seeing the clients for a very short time, 1 afternoon every 10-12 weeks, so we give them as much information as we can, especially when we are saying to them, look, we don’t think this resident xx requires this amount of haloperidol. If we have got the evidence, they are much more inclined to take our word for it, listen to our point of view. N3 |
| 10.2  Man, 95 | Aggression and incontinence disappeared when antipsychotic discontinued. | *Nurse completed ADRe, and used it as evidence to persuade GP to discontinue antipsychotic. Promazine was stopped, and within a month a*ggression and incontinence were no longer problems.   1. caviton barrier cream as directed, 2. lactulose 7g/5ml oral soln, 3. promazine 25mg/5ml oral soln 2.5 ml bd as req, 4. Resourse Thickenup clear powder 6 times / day 5. Clotrimazole cream 1% 2-3 x day to gentitals, 6. Paracetamol stat doses for pyrexia or pain | 1. Cognitive decline, 2. behaviour problems, 3. physical violence, 4. aggression, 5. confusion, 6. incontinence, 7. snacking, 8. low vit D intake, 9. swallowing difficulties, | [He was] much brighter. He was quite drowsy when he was on the medication. But once we’d convinced the doctor, (laughs)..if that’s the right word, that he really didn’t need it and we could manage his behaviour and his night rotation, he was actually all right & he settled, there was no problem, so he didn’t need the medication after all. N10 |
|  | **Barriers** |  |  | Articulation of the problem |
| 2.2  Woman, 63 | **Time for nurses and doctors**  Despite lack of time, 2.2’s  convulsions were reported to prescribers | The only records of seizures were on ADRe. ADRe documented that seizures were occurring, and this was used to report to prescribers. We noted that levetiracetam might be contributing to seizures as well as insomnia, eczema, irritability. However, no changes were made.  Vital signs not recorded, and lack of time was given as the reason.   1. adcal d3 chew tabs 1bd, 2. alendronic acid 70mg once week same day, 3. forceval caps od 1 hr before meal, 4. levetiracetam bd. Dose illegible. | No vital signs, qs on falls, headache, tinnitus, paraesthesia, sob not recorded – ‘doesn’t talk much’  Noted:   1. Feet shuffling, 2. bruising, 3. stores food mouth like gerbil, 4. doesn't listen, 5. cognitive decline, 6. irritability, 7. hyperactivity, 8. mood fluctuations, 9. insomnia, 10. convulsions, 11. behavior challenging. 12. Rash ?itching - scratches, 13. misses meals, 14. snacks, 15. vit D, 16. sugar, 17. no dark glasses | There is no possible practical way that one is going to get to see senior medical staff and prescribers: it’s just not going to happen. The psychiatrist relies very heavily on what the care staff are saying. This might strengthen what the care staff are able to say about medicines impact, and we can only react positively. SU 2  Confirmed by nurses:  You’ve got to produce the evidence to the GP and this is ideal to produce the evidence. N2  I don’t think GPs have got the time. They do their best while they are there, but it’s not routine to check on every patient regularly. SU8  In the cut & thrust of their daily routines it will be very easy to get lost. SU2  All the questions are important, but it does take time. If it could be condensed, somewhat, there would be better compliance because it takes a fair while to take all that in. You’re looking at dietary intake, blood results and all that. I’m not saying it’s not important, but if this is to roll out and be something everybody should have on their file, regular medication review and assessment. It needs to be more time friendly. Nurses and doctors just don’t have 45 minutes to fill in a form. If you’re trying to get blood results, you’re ringing the surgery, can’t get through, can’t get the results. (…) It would have to have time dedicated to it. N6.2 [ blood results are to be supplementary only]  The biggest barrier is going to be time, but having said that, once you’ve used the tool & you’ve used it regularly, it becomes second nature, so it’s not that time-consuming. It’s like everything else really, slow at first and use it regularly and get quicker. N10/ N1 similar  We observe and we are busy as we have 91 patients. nurse time is the hurdle and getting staff on board. If staff are responsible for 21 patients, then there is time pressure. N7  The net workload is probably closer to zero or maybe positive. If you were able to more consistently identify patients who were being aggressive or violent or irritable and manage that effectively, you would reduce your workload in the longer term- you would have less incidence of those things and improve the management of continence. S1  Given how much pressure they are under and what their workload is, I think they would struggle to have the time to complete this [ADRe] as well. GP1 / P1 similar |
| 1.1  Man, 66 | **Fit with other documentation** **/ resistance to change** | Vital signs results and ADRe were passed to GP. The patient was referred to the GP for full medication review (over a year since last review) to address hypoxia, hypotension, dyspnoea, absence of constipation (laxative prescribed). The nursing home enhanced their monitoring. Prescribed:   1. paracetamol 500mg PRN (1-3/week given) 2. amlodipine 5mg mane 3. Aspirin dispersible 75mg mane 4. Dipyridamole MR 200 mg bd 5. gliclazide 80mg mane 6. laxido 13.8g bd 7. quetiapine two 25mg bd 8. venlafaxine MR 75mg mane 9. solifenacin 5mg mane 10. simvastatin 10mg nocte   inhaled   1. Clenil modulate (beclometasone) 200mcg bd inhaled 2. Ventolin evohaler 200mcg qds inhaled | 1. oxygen sats 93% to alert to sob etc 2. Catheter present 3. ADRs of antipsychotic e.g. weight gain, hyperphagia, restlessness 4. BP from Profile, not otherwise done 5. Postural hypotension 158/92 & 126/75 6. behaviour, 7. self-harm, 8. agitation, 9. restlessness, 10. moods, 11. insomnia, 12. catheter, 13. sob, 14. appetite, 15. rash, 16. snacking, 17. sugary drinks, 18. vision, 19. sometimes feels the cold | We do monthly observations regularly, and as and when. So it’s like duplicating what we already do, but it is more in-depth. (…) it has highlighted some of the things that you wouldn’t think about asking, like dry mouth, gait, the physical side effects. (…) there are some things that I would never have thought to look for - tongue movements. N1  It’s another piece of paper, and there’s enough. If you’re going to do these things, like the paperwork, you’re taking the carer away from their roles. (…) There are a fair few carers who just raise their eyes and say “Oh no, not something else”. (…) So integrating this into routines, looking at whatever else they are monitoring with a view to keeping it manageable and not distracting them from interaction with residents. Su2  Anything new upsets the apple cart N10  People won’t write it twice. (care home is electronic records only) The questions that you have here, they are good because they would make that nurse look to the files and to actually acknowledge exactly what that person had for food that day. The idea behind it is good. The only problem is we already have a system in place. You’ve just come in with another piece of paper for us to fill in you know.(…) . But the profile is very good because it will remind people of the basics of nursing and what to actually look for. (…) Because if I’m going to ask the doctor to come and see one of my patients, I just give him the basics and why I need him to see him but it won’t be as detailed as this. This will make a lot more people understand a lot more things about how the person behaves. How that person is. So, the profile is good N8  Contrast  Today we have to document everything. That’s part of working in this environment. But, it is a very useful tool … it’s easy … it’s all there. You know what happened, with the BP and all the things like that as well. They (doctors) can look at that, then it’s the doctor that makes the decision. That’s why we use the profile. N3 |
| 5.1  Woman, 83 | **Prescribers** | ADRe identified chest pain, dyspnoea and insomnia. However, there was no response from the GP.  The pharmacist indicated that sleep would be improved by moving promazine to morning administration, reducing doe of chlorphenamine to within BNF guidelines (from 16 to 12mg/ day), discontinuing ineffective hypnotics and reviewing salbutamol nebules and inhalers as indication was not clear.  An optician’s appointment was made.  1. apixaban 2.5mg1 bd,  2. bisoprolol 5mg od, 3. digoxin 62.5mcg od,  4. furosemide 40mg 2 mane &1 lunchtime (total 120mg sj),  5. omeprazole 20mg gastro resis caps 2 mane,  6. prednisolone 5mg od mane,  7. chlorphenamine 4mg 1 4-6 hrly as needed for itching,  8.co-codamol 8mg/500mg 1-4 x daily,  9.dermol 500 lotion, 10.diprobase 3-4 x daily,  11. eumovate0.05% night thin apply,  12. laxido orange oral sugar-free 1 sachet bd, 13. oramorph 10mg/5ml oral sol half a 5ml nocte, 14. 14. 14. promazine 25mg/5ml oral sol 2.5 ml evening  15. salbutamol 2.5 mg/2.5 ml neb liquid 2.5 mg bd  16. senna 7.5mg 1 or 2 nocte  17. temazepam 20mg nocte when necessary  18. ventolin 100mcg EVOhaler 1-2 puffs up to 4 times daily when required | New problems were identified ‘You didn’t tell me’/ ‘Why didn’t you mention this?’ ‘If I came to the nurses every time …’   1. Hand tremor, 2. feet shuffling, 3. bleeding from scratching, 4. feeling cold, 5. irritable, 6. agitation, 7. mood fluctuations, 8. insomnia, 9. sedation, 10. tingling in arms, 11. pain in L breast/ chest pain, 12. short of breath 13. appetite changes, 14. rash itching, 15. oedema, 16. missing meals, 17. no milk, 18. no vegetables, 19. indigestion, 20. dry mouth, 21. vision problems, 22. no dark glasses, 23. pain, 24. tablets crushed | What I found here is that the GPs are not very forthcoming. Um, it’s like today, the phone call that I got from the doctor with reference this lady, I had to make a week ago, so to me that’s not good enough, really. You’ve got to book a telephone consultation, if you want to discuss someone’s medications or to get advice on medication. You speak to the receptionist that you want to speak with the GP with reference bla, bla, bla, you could be waiting two- three days. Then you get frustrated yourself really. (…) ADRe picked up 2 issues. Like I say, I spoke to the GP, they weren’t forthcoming in helping, but they shut me down, really. N5  The resident corroborated the nurse’s request for additional input:  The tablets I’m taking, I don’t know what they are for. Bed time tablets, I take them between 9-10, I go to bed and I’m awake between 1 & 2. I’m taking sleeping tablets & I can’t sleep & oromorph – I’m still not sleeping. Su 5.1  My experience is that pharmacists are keen to come on board, the consultants too. GPs are a difficult group. (…) they always think they know best. (…) we all know that Joe Bloggs doesn’t need the risperidone, but the GP thinks he does, so it’s getting round that sort of thinking. N10  Resistance from the GPs because they might feel threatened, they think that nurses shouldn’t be having any input – they know best. N10 |
| 7.2  Woman, 62 | Nurse complacency / entrapment by prior expectation | ADRe recorded oxygen saturation 94%, BP 109/75, HR 104, posture abnormal, dry eyes, unable to stand. 7.2 was hypotensive and taking amlodipine (presumably for hypertension), furosemide 40mg, losartan, baclofen, oxycodone, doxazosin. Tachycardia indicated baroreceptor reflex activated & hypoxia suggested reduced tissue perfusion, possibly linked with hypotension. No action taken.   1. Adcal-d3 chew tutti frutti, 1 bd, 2. amlodipine 5 mg 1/day, 3. baclofen 10mg tds., 4. doxazosin 4mg mr 2 od, 5. fluoxetine 20mg 2 mane, 6. furosemeide 40mg mane, 7. gabapentin 300mg 3 tds, 8. letrozole 2.5mg 1 mane, 9. omeprazole 20mg 1 mane, 10. senna 7.5mg 2 nocte, 11. diprobase cream PRN for dry skin (not given), 12. Longtec (oxycodone) 40mg bd, 13. mometasone 50mcg nasal spray as directed, 14. nizoral 2% shampoo PRN, 15. paracetamol 500mg 2 qds, 16. viscotears 2mg/g gel as directed 17. Losartan 50mg, 1 daily | 1. Oxygen sats 94%, 2. BP 109/75, sitting, unable to stand 3. tachycardia 104, 4. temp 36.1 5. posture abnormal, 6. dry eyes | Unlikely the medicines will be changed. I am sure they won’t be changed. N7  We get used to that person, so we might omit some signs that we don’t actually think are noticeable (…) the profile will ask you different questions that maybe you never thought of. (…) you might see but might classify that as age. You might not actually put it down to medication. (N6)  It’s difficult to know what’s a side effect and what’s the disease. SU4. |
| 5.3  Woman, 81 | **Overwhelmed by the system / defeatism** | ADRe identified pain for the first time, and the nurse reported to GP, seeking stronger analgesia and a medication review. However, there was no response.   1. Adcal-d3 750mg/200 units 1 bd, 2. levothyroxine sodium 100 mcg od mane, 3. paracetamol 500 mg 1-2 q 4-6 hrs as required, often given 4. clopidogrel 75 mg od, 5. memantine 20 mg od, 6. monomil xl 60 mg half tablet daily, 7. laxido orange oral sachet sugerfree 2 sachets bd as req, 8. mst continus 10 mg bd, 9. oromorph 10mg /5 ml sol half a 5ml spoonful when req, max 4x a day, marked NT bd 10. sertraline 50 mg od, 11. thiamine 100mg 1 tab bd, 12. alendronic acid 70 mg 1 tab once week, 13. diazepam 2 mg 3x day when req, seems to be given nocte only 14. glyceryl trinitrate 400 mcg/dose pump sub ling spray as needed- not given recently | The resident was chair-bound, unable to stand, and had very difficult speech.   1. weight gain 1.5 kg, 2. BP 103/58 3. posture, 4. bent over, 5. gait, 6. balance, 7. feels cold, 8. cognitive decline, 9. behaviour, 10. physical violence, 11. verbal aggression, 12. irritability, 13. anxiety, 14. restlessness, 15. confusion, 16. daily mood fluctuations, 17. insomnia due to pain, 18. incontinence, 19. constipation, 20. misses meals, 21. pain,   message to prescriber: pain | To me personally, it feels like they’re old, they’re stuck in a care home, what more can they do. That’s how it comes across to me. They put them in a care home & forget about them. (…)The majority of the residents have been here now a few years and it’s quite difficult to start messing around with their medication.N5  A lot of people are on a lot of drugs that aren’t reviewed. I1  A consultant explained how antipsychotics can sometimes be the only realistic option: Behavioural or psychological symptoms of dementia, if the antipsychotic, even at a very low dose is withdrawn, the placement breaks down. That’s to do with agitation, restlessness, the case mix in the home (if they have 4 or 5 people needing attention that’s okay, but if everyone needs attention then it’s the straw that breaks the camel’s back). We’ve found that though it’s possible to stop antipsychotic medication and to identify when it’s not absolutely necessary, the fact is that the care home placement will breakdown and we perceive a small dose of antipsychotic medication supervised as being preferable to a hospital admission or a change of placement. |
| 2.3  Woman, 69 | **Staff turnover / education** | The pharmacist recognised that beta blockers were causing the resident to feel cold. Blankets were provided.  Magnesium hydroxide was prescribed to a resident with diarrhoea.  These problems could be addressed by using the supporting information to educate nurses or carers. Prescribed:   1. epilim chrono 300mg 1 bd, 2. propranolol 40mg 1x3 day, 3. buccolam oromucosal sol 2ml as needed, 4. Magnesium hydroxide mixture 2 x 5ml bd. | 1. feet shuffle, 2. cold feels, 3. balance, picky, 4. verbal aggression, 5. irritable, 6. seizures/epileptic, 7. low energy, 8. restless, 9. agitation, 10. behaviour problems, 11. mood fluctuations, 12. sleep disturbed, 13. sedation, 14. discomfort on urination, 15. diarrhoea, 16. vision problems, 17. no dark glasses, relives bereavement, 18. cognitive decline, 19. lactose intolerant, has own milk, forgets she has eaten, 20. doubly incontinent | If you’ve got a whole shift system of quite considerable numbers of staff dropping and changing like this, it’s about the practical dangers of not being done consistently. (…) who carries the responsibility for making sure this is happening and making observations. SU2 offers to complete ADRe I wouldn’t find it burdensome.  We don’t get a lot of information from prescribers. Communication is only if we cannot get the medicines. (…) Lack of time for all professionals. (…) We give medicines with food, not on an empty stomach. N7 (this reduces the effectives of many medicines, including anti-bacterials, and *post cibum* should only be for certain medicines.)  We need a national review of social care (…) to try to give some recognition to care staff. It is very hard work, and very often care staff feel “I am only a care assistant – but no, you are not *only* a care assistant – it’s a really important job and lots of people couldn’t do it. (…) Some homes are really struggling. The staff are doing their utmost, but when you haven’t got the support from management everything is very difficult. When you have a high turnover of staff or you are using a lot of agency staff, they don’t know the clients. They are not able to fill in the paperwork. They just come in and do their shift and are very concerned with just doing their shift. They haven’t got time to look at profiles and documentation. That is the reality, there are not enough staff. Nurse consultant |
|  | **Facilitators** |  |  |  |
| 9.2 woman, 69 | **1 to 1 time** | Knee pain identified and responded to paracetamol administration, which was available. Diagnoses were psychosis & recurrent depression. Only prescription was aripiprazole 10 mg 2 each day. | 1. Girth 108 cm, 2. Cognitive decline, 3. R knee pain, 4. anxious, 5. confusion, 6. headache 7. snacking, 8. sugar in drinks, 9. no sunscreen 10. or dark glasses | One of the residents we spoke to she has pain in the R knee. She never told anybody, when we do medication, she never mentioned that before. We can use our home remedy, paracetamol N9 |
| 4.1  Woman, 82 | **Interpersonal relationships** | 4.1 was pleased that nurses were taking time to check, felt reassured, and enjoyed the attention.  Medication review was arranged. Risperidone dose was rather high for a small person, and could be linked with shuffling, restlessness, cognitive decline, hypersalivation.  Postural hypotension was recognised, and marked for monthly review (risperidone, timolol, sertraline).  Falls risk to be monitored, but not a current problem.   1. omezprazole 20mg, bd, 2. risperidone 1mg -nocte, 3. risperidone 500mcg bd 4. sertraline 50mg- 1 mane 5. trimethroprim 100mg- 1 nocte 6. brinzolamide 10 mg/ml/ timolol 5mg/ ml eyedrops. 1 drop bd, both eyes, 7. latanoprost 50 mg/ml eyedrops- 1 drop at night- affected eye. | Body weight 55kg.   1. feet shuffling, 2. feeling cold, 3. cognitive decline, 4. agitation, 5. restlessness, 6. confusion, 7. nocturnal incontinence, 8. hypersalivation,, 9. diarrhoea, 10. worsening skin rash, 11. snacking, 12. denture problems. Staff were not previously aware that dentures were loose. | We are told about what’s going on. We visit regularly (…) we can always talk to staff. They [care staff] check regularly, but if that can be improved then this is good. Su4 |
| 10.3  Man, 78 | **All information in 1 place** | Liaised with doctor to reduce and remove diazepam. This removed over-sedation & confusion.  MAR chart indicated diazepam 2mg tds (discontinued)   1. Resourse Thickenup, clear powder 6/day 2. midodrine 5mg tds 3. zopliclone 3.75mg nocte, (not given) 4. cavilon durabe barrier cream as req- not in cassette, 5. conotrane cream as directed- not in cassette, 6. fucidin 20mg/g cream 3-4 x day lesion on head, | 1. Shaking at rest, 2. sedation improved, 3. urination problems, 4. lack of milk and vitamin D, 5. sleeping interfering with intake, 6. dry mouth.   Better when diazepam stopped.  No lab tests done. | Without using the profile, we tend to find GPs would prescribe mental health medication that weren’t really appropriate. ADRe identified, you didn’t really need these on a regular basis: PRN or not at all. So for all of us at H10, it did identify that we needed to be more in contact with the GPs & say, look you know, this isn’t working. This person doesn’t need to be on risperidone etc. You can distract residents they’re much more settled without risperidone. That’s what we found. N10  All the information on 1 piece of paper, it’s good for the residents, rather than going through separate pieces in separate places. (…) If the doctor comes in and says “Have you done the observations?” it’s all together in one place and you can give the details to the GP. N9  All the information in 1 place allows you to focus on the person. (…) I did the profile myself, I did the vital signs, wrote them all down and I looked through all this to see any of the symptoms, ticked them off. (…) it was their agitation. I discussed with the GP then. N2.  They [doctors, pharmacists] don’t look at the assessments and care plans and things that we do because it’s just mainly for the local health board and for us to have, in order for us to handle the residents better. N8  All information in 1 place: this is bringing together assessments you are probably doing already, but bringing them so they have a focus around medicines. S2  Everyone is aware of what issues are coming up with the patient, and the decisions can be made between all the healthcare professionals. P2 |
| 8.1 Man, 89 | **Identifying unrecognised problems** | Pain was recognised and treated with paracetamol. Diet diaries were completed to assist weight gain management. Carer felt ADRe identified too many problems to write them all into the care plan, but found ADRe a good opportunity to get to know the resident.  The pharmacist identified a number of possible causes for the tremor and falls: mirtazapine, memantine, tamsulosin, diazepam, venlafaxine, quetiapine. Prescribed:   1. tamsulosin 400mcg, 1 mane, 2. metformin 500 mg 1 tds, 3. simvastatin 20mg 1 night, 4. lansoprazole 15mg mane, 5. levothyroxine 50mcg mane, 6. clogidoggrel 75mg 1 mane, 7. allopurinol 300mg 1 mane, 8. diazepam 2 mg half tab qds, 9. venlafaxine 75mg 1 mane, 2 night 10. quetiapine 150mg 1 night, 11. paracet500 mg 2 4x day, CHECK 12. laxido orange 1 2x day, 13. lactulose 3 5ml 2x day PRN, 14. mirtazapine 45mg 1 night, 15. memantine 20 mg 1 mane, | 1. Wt seems to be a problem, but without height we can’t assess. 2. hand, 3. tongue tremors, 4. feet shuffle, 5. abnormal movement, 6. gait, 7. posture abnormal, 8. balance, 9. feel cold, 10. cognitive decline, 11. dizzy, 12. behavioural problems, 13. headaches, 14. violence, 15. aggression, 16. irritability, 17. hallucination, 18. agitation, 19. sleep problems, 20. restless, 21. confusion, 22. mood, 23. apathy 24. halitosis. 25. falls, 26. tinnitus 27. urine problems, diabetic, hair loss 28. missed meals 29. snacking 30. denture problems 31. vision problems   patient requested medicines review | We get used to the person. We might omit some signs that actually we don’t think are noticeable… need to take notice when you look over the profile it will ask you different questions that maybe you never thought of. Someone being on a different kind of tablet might give them tremor. You might see that but might classify that as age. You might not actually put it as medication. So this is why the profile is good. And maybe sometimes we need to remember the basics of nursing. Some of us have been doing it for so long…either we forget or we make mistakes. Then what’s actually what’s more obvious than what’s in our face what’s in front of us? N8  Similarly:  I made notes on the profile, to lead me in the right direction. Rather than just trying to put my opinion across, I had the evidence in front of me: look this is her BP, her falls have increased – four times more. Having the directions [supporting information] to say “it might be this medication, so can you come and review?” It’s useful but quite complex. N6.2 |
| 3  case from interview | **Staff feel valued** | For another lady, who was with us when we initially did the first Profile, [in 2013], ADRe identified quite a number of issues: we found she had anaemia actually, which hadn’t been diagnosed. She was quite agitated. She had a rash that was itching and things, which contributed to her mood and was making her irritable and things. When we used the Profile, we found she was prescribed diazepam and it had no effect at all, and she was also prescribed risperidone, and again that had no effect. It wasn’t actually her mental health, but she had anaemia and that was causing [problems]. Her blood pressure was quite low, she wasn’t eating and drinking very well-she was under 50 kilos. We found, when she had some bloods done, they found her Hb was low, she had pernicious anaemia. She has some injections and her weight went up. She did improve significantly and that was identified through using the Profile. | This late resident was not enrolled in the research project. | What it [ADRe] tends to do, is give value doesn’t it? The staff-senior staff tend to feel the information that they are gathering is of value. It improves moral. The care staff – the staff feel that what they are doing is worthwhile and they are not just ticking forms, ticking boxes and just getting filed away. N3 |

Note: key sentiments have been highlighted.

### Table C. Changes needed to reduce ADRs

| **Change** | **For** | **Against** |
| --- | --- | --- |
| **Supporting care home staff across the sector** | | |
| Information availability | I have asked for an up to date BNF, I’ve been told there is one somewhere but I can’t find it. (…) the GP should be more forthcoming with information. N5  Do this regularly, because it reinforces that learning and is more likely to embed a general consideration of these issues in their day to day work. |  |
| Staff education and literacy | Two of the care homes that dropped out of the study stated that the care staff were unable to understand ADRe and the supporting information.  It [ADRe] may be too difficult for them [staff]. Gp1  Simplify profiles, seems very complex, too much information. N7  I’ve shown it [ADRe] to [non-registered] staff and they’ve gone “what’s all that?” I think they feel it’s too much. (…) I know we should be doing this routinely. It’s just getting people to take it on board. It’s trying to find the time to sit and do this, when you’ve got so much other things going on. N2.  The care home staff are very variable – the most fantastic people I know – some are not desperately well educated, clearly have difficulties reading and writing. It is clearly a problem. I don’t think we should underestimate what a challenge this would be. SU2 | Many carers did not have problems:  [The supporting information] the carers understood it, so they got the knowledge, and what to look for and the explanations. N10  It [ADRe] was quite easy N9.  ADRe was rated as ‘easy to use’ in 27 of 30 instances. |
| Care home staffing | If a HB is commissioning continuing NHS care they have to make sure that there is sufficient nurses to care, and within that, I would expect there to be enough time to be able to manage medicines, and look for side effects and ongoing monitoring. S2  When you start using it [ADRe], it should be much quicker to do. They would need to be resourced for it – the time. They have got lots of things to fill anyway. The ‘No Tears’ tool depends on the person’s knowledge of the drug and what you should be looking for – it’s very easy to miss something. This one covers everything, but becomes lengthier. You won’t miss anything here, but administering this would take more time and resources. GP2  It is going to have to be thorough. (…) your main obstacle again is going to be time. With the drugs as they are anyway, this is going to add….for 42 patients, you are going to have 2 a day, it is going to add 40 minutes to their drug round so that is going to be one issue for them. The nurses might have to go through extra training as well. P1 | Care services’ failures are often due to an inability to sustain staff teams, a lot of agency staff. A lot of the errors with medication and a lot of the problems with medication arise because of agency staff. We see CHs going down and down and they enter a dangerous cycle where they begin to fail, because they have an embargo that means less income, less income to employ staff, staff start walking because they feel it’s unsafe -they don't want to work there and it becomes very difficult. I1 |
| **Standardised prescriber availability** | | |
| GP contact / communication | The GPs say we’ll put them on such and such. They don’t tell you what it is for. They won’t tell you any side effects. If they put them on antibiotics, for instance they just write the prescription up and you can guarantee about an hour or two later, you’ll get a phone call, saying this you’ve got to stop this medication because it counteracts with the antibiotics. Well, to me, surely, they should tell you that as give you the script, especially as it’s a home visit. It would be helpful. N5  The side effects of the medication that I’m currently taking I’ve only found out by looking at the internet (…) There was no follow up and even though my GP was fantastic (…) if you take a step back, he has got a corridor full of children with tonsillitis, pregnant mums, people suffering with cancer. (…) my diabetes is monitored constantly every 6 months, but the mirtazapine has never been. SU W (note, mirtazapine is associated with weight gain and diabetes [Siafis & Papazisis 2018]).  GPs and even consultants, they prescribe medicines without a very close touching base of what the impact is. SU2  Message to prescriber “Do your job. See us when asked”. SU 5.1  The GPs don’t actually listen to what we’re saying, they tend to talk to the service users, who can’t answer and will agree to anything. They’ll say “yes”. We say “no, no, you need to talk to us as well as the advocate”. They don’t always listen properly. (…) I understand that they’re busy, they’ve got so many patients. (…) You can fill in the profile and fax it through & they’ve got all the information in front of them. N2  We know the GPs should be doing annual reviews, but it doesn’t always get done. N7  There are problems between GPs and care home staff. (…) several instances where we asked for a medicine to be stopped, and it wasn’t. A patient had kidney problems and we asked for 1 of their blood pressure medicines to be stopped and when we returned 2 weeks later it was still ongoing and the kidney function had deteriorated. GP1 | Some homes had no problems:  GPs … they come straight away. If we’re not sure, we ring the surgery again and ask. Communication will be there all the time, so we don’t do anything wrong with anybody’s health. We ring the out of hours doctors and they will advise us. N9  We have a close relationship with the GP [name]. I have been here a long time, she has been in the surgery a long time. Somebody on a syringe driver, medication was incorrect; the staff picked it up straight away (before administration). N3  We have annual meds review GP2  GPs don’t have a lot of time. P1  I’d say the relationships between GP practices and pharmacies are far better than they have been for a long time (…) everybody lives with the fear of litigation as well as patient harm. P3  GPs are working alongside pharmacists and appreciate their skills more than they’ve ever done before. P4. |
| Consultant contact | Antipsychotics, you shouldn’t be on them very long, cos of the side effects, but nobody seems to take any notice. So, I asked to see the psychiatrist. It took me months and months. I caught a glimpse of her in the corridor and she kept promising that she would make an appointment, but she was busy. The GP was unable to lower the medication. SU8  note Risperidone for dementia symptoms licensed for 6-8 weeks.  The nursing staff, I think they have difficulties, and behind that there is the psychiatrist, and I am not entirely convinced that he knows what’s what, so it’s a problem. (…) J was having seizures, the staff were worried, and it must have taken 6-7 weeks to get a consultant response via a GP. It put the home management under a lot of stress and cost them time. SU2  Wasn’t good enough SU 10 | Homes 3,4 and 9 reported good consultant liaison:  Doctors will always ring back the same day. (…) 4.3 there were a lots of inconsistencies, his sleep, diet, very unclear what the changes were. By using ADRe and his dribbling [on risperidone] the MH team were involved and changes were made for the better. We’ve lost 1 of his medicines in the day and he’s now more awake as a result. N4  Getting hold of them, their workload (…) the team come to us every 12 weeks, used to be every 8 weeks. N3 |
| **Expanding pharmacists’ roles across the sector** | | |
| Pharmacists out of the loop / no  contact | We never see the pharmacists. They send the prescriptions to the pharmacy. We should be in more contact with nurses or doctors, to find out what tablets we’re on and what they are for. We can’t sit here and be dished out a load of tablets and not know what they are for, but that’s what we’re doing. Su 5.1, 5.2  Care homes don’t tend to ring us for a huge amount of advice about some of the medications- they are quite just GP happy- they do just tend to go straight to the doctors and they do tend to get prescriptions that way, because they have, probably like daily visits from the GPs, about various other things P1  With GPs, depending on the GP....it can be quite hard to track them down sometimes. You ring the Surgery and the Dr is out on home visits-can we get them to call you back? That’s just the nature of GPs. I think-they say they are out on home visits, they don’t want to be disturbed about calls about other things unless it is an emergency P1  The homes go straight to the Dr. They tend to get in touch with us if they can’t get hold of a Dr in a reasonable amount of time. (…) WE have no direct line of communication with GPs. Some surgeries are extremely difficult - to get the receptionist to request a conversation with the GPs - a major barrier. When you can get hold of them, they are fine. P2  I have extremely little input when it comes to the management of the medication, which is unfortunate because I do have, and all Pharmacists have, a lot of knowledge when it comes to the medication. The people working in the CHs are actually thinking to come to us, rather than going straight to the GP. It might be a case of educating them that we are the medication experts, and if they need help, to come to us rather than the GP. P2  There is potential for pharmacists to do much, much more. P3, P4. |  |
| **Reflect before repeat** | | |
| Medication Reviews | I’ve never been approached by any of my medical team, with regard to coming off or not coming off or tailoring the dose. It’s just I was put on that dose and that’s what it’s been ever since [6 years ago] (...) you see Dr. X, the last time was 3 years ago. SU W  GP surgery, there’s a lot of complacency with the staff, it’s that conveyor belt. Prescription in, prescription out (…) when I hand the prescription in, the girl is not looking, she doesn’t know why I’m taking that. They call your name, you get it and off you go. I’ve never had any communication from them. They could take a few lessons in retail. SUW  I don’t think they [medicines] are being checked enough SU 5.1, 5.2  Medication should be reviewed without some relative having to press for it. SU8  I don’t know whether there was any space on those [MAR] sheets to record adverse effects. (…) Mediation review required because some had fallen (…) the resident was unsettled or displaying symptoms that were difficult to manage. GP1  Half the time we give medication to stop the side effects of another medication and we don’t think about what the benefits and harms of each of these drugs are given patients’ frailty. GP1  There isn’t enough attention, enough confidence about withdrawing drugs. People tend to create scripts and let them run and are anxious about taking them off. Often people are admitted to hospital and the first thing they do is take them off all medication and their wellbeing can improve. I1  You can prescribe patients all sorts of medication but if you are not following up on whether they are getting side effects or whether it is actually therapeutic…. there is no point in giving it in the first place. Simvastatin for example, is really common with muscle aches and pains and if that is the case and the patient is suffering from that…then… that is not an effective method of treatment and that should be switched. P1  The homes just send us the prescriptions, they won’t tell us why they are on the medication … you have to ring the doctor. P1 [nurses did not appear to know the indications for the prescriptions.] (…) I wouldn’t say communication between the GPs, Pharmacists and the CH is especially great, well, in this area anyway. P1 | There are definite concerns about the consistency of what is done, the quality with which that review is undertaken. (…) A lot of medication reviews will be paper exercises or desk-top exercises with a computer looking through people’s records without engaging the user in a discussion about what is right for them. (…) if a GP or pharmacist undertaking a review were able to see all this information whilst they were doing the reviews, it would significantly improve the quality of them. Stakeholder 1 |
| **Adopt ADRe** |  |  |
| ADRe | This is spot on. (…) it’s the fact that people are stuck on medication, pigeon holed and left. “Just keep taking the tablets.” It’s important that reviews happen, and that is why I think this is good – it highlights something that can be improved upon. SUW  Information about patients’ medicines (…) there could be more, in depth and they don’t give us enough on what the side effects are and what to look for. (…) [ADRe] it’s in front of you, so it makes you aware of what to look for. We can ring them (…) pharmacists and prescribers don’t look at the nursing notes. N2  It would be nice to have one in every set of notes. A checklist, same as every other document we’ve got, Waterlow score, weight chart. Physical paper would be quicker and accessible. We need close guidelines on as required medications. N4  What the profile would appear to do is bring about a much greater awareness of the problem and a much greater consistency in the approach to dealing with it. (…) highlights the most likely problems and the guidance helps people interpret what they might be seeing when they identify one of those problems. But, everyone is busy. (…) the profile would be a great benefit to all homes, making sure the ones who would benefit most are the ones that adopt if and adopt it sooner rather than later. S1.  The GPs are prescribing the medication and are also commissioned through the enhanced service agreement to do a medication review – why would they not be doing this? S2  good, comprehensive, doing ECGs and all that GP2  meeting a need / filling a care gap  I don’t think there was anything in place to prevent them [side effects] occurring. it was all very responsive, rather than planning for it. GP1  We have a lot of questions, social contact, hygiene, infection control, preparation of food, nutrition, it goes on and on. Amongst that you would expect a list about medication. The regulations that we work with are about the safe administration of medication – not proposing the best use of medication to promote wellbeing. So long as a home has a prescription, receives the drugs, can show its audits and things are administered we are happy. Whether the medication is the right medication is not our business. I1  We would certainly see things like this as good practice markers. If a home could evidence. I think the question we are trying to answer here is - are the people with the day to day care in the home - are they sighted in all the subtle signs that may exist and may point to problems with medication and any tool that can help with that - this is a good tool - it has been well thought through. The regulations at the moment don’t require that. The regulations are very much about- in terms of medication, safe administration, what this goes a bit further to do is to deal with that part of the regulations which is about promoting health and well-being. I1  Just brilliant, extremely straightforward, self-explanatory, easy for someone to fill it out. P2. | It [health board] can be slow to respond, the committee and the introduction of new medicines, that’s well supervised, but existing medications and particularly the relatively dull routine maintenance of ordinary medication – I don’t think anybody does that. Consultant.  You wouldn’t choose to regulate for people to do this until people were doing it voluntarily, and it was part of practice in a lot of homes, gives you confidence to mandate use. (…) Someone will come with a clipboard and audit that it is done rather than caring about why it is done with a focus on the outcomes it could deliver. Stakeholder1  Sometimes you get ritualistic compliance. The more questions you ask, the more burden you create and the less thinking you do. (…) charts and things being ticked but the care not being delivered. I1  Nocebo effect / acquiescence response  Leading questions, because the patient is more likely just to say oh yeah I do have that or whatever. There are like a yes, no, worse answer. If you are asking some patients do you get dizzy or light headed-they might just say yes. P1 |
|  |  |  |

Note: key sentiments have been highlighted.
